# Interventions to Address Potentially Inappropriate Prescribing for Older Primary Care Patients: A Systematic Review and Meta-Analysis

**DOI:** 10.1001/jamanetworkopen.2025.17965

**Published:** 2025-06-27

**Authors:** Nav Persaud, Aine Workentin, Amal Rizvi, Tiphaine Pierson, Émilie Bortolussi-Courval, Kathy Liu, Alexandria Bennett, Nicole Shaver, Becky Skidmore, Niyati Vyas, Robert Pap, Faris Almoli, Todd C. Lee, Caroline Sirois, Rita K. McCracken, Louise Papillon-Ferland, Emily G. McDonald

**Affiliations:** 1 MAP Centre for Urban Health Solutions, Li Ka Shing Knowledge Institute, St Michael’s Hospital, Unity Health Toronto, Toronto, Ontario, Canada; 2 Department of Family and Community Medicine, University of Toronto, Toronto, Ontario, Canada; 3 Department of Family and Community Medicine, St Michael’s Hospital, Toronto, Ontario, Canada; 4 Canadian Medication Appropriateness and Deprescribing Network, Montreal, Quebec, Canada; 5 Centre for Outcomes Research and Evaluation, Research Institute of the McGill University Health Centre, Montreal, Quebec, Canada; 6 School of Epidemiology and Public Health, Faculty of Medicine, University of Ottawa, Ottawa, Ontario, Canada; 7 Skidmore Research & Information Consulting Inc, Ottawa, Ontario, Canada; 8 Clinical Practice Assessment Unit, Department of Medicine, McGill University, Montreal, Quebec, Canada; 9 Faculty of Pharmacy, Université Laval, Québec, Québec, Canada; 10 Department of Family Practice, University of British Columbia, Vancouver, British Columbia, Canada; 11 Faculté de Pharmacie, Université de Montréal, Montreal, Quebec, Canada; 12 Research Center of Institut Universitaire de Gériatrie de Montréal, Montreal, Québec, Canada; 13 Division of General Internal Medicine, Department of Medicine, McGill University Health Centre, Montreal, Quebec, Canada

## Abstract

**Importance:**

Prescriptions for potentially inappropriate medications are common and, by definition, may carry risks that outweigh benefits.

**Objective:**

To determine whether interventions to address potentially inappropriate prescribing for older primary care patients are associated with changes in the number of medications prescribed, drug-related harms, hospitalizations, and mortality.

**Data Sources:**

MEDLINE, Embase, and the Cochrane Central Register of Controlled Trials were searched from inception to September 6, 2024.

**Study Selection:**

Randomized clinical trials of interventions to address potentially inappropriate prescribing for older primary care patients (aged ≥65 years) residing in the community or in long-term care facilities, such as nursing homes or assisted-living facilities, were included.

**Data Extraction and Synthesis:**

Two researchers independently screened the records and abstracted data using the Preferred Reporting Items for Systematic Reviews and Meta-Analyses (PRISMA) reporting guideline. Data were pooled using random-effects models.

**Main Outcomes and Measures:**

The planned outcomes were the number of medications, nonserious adverse drug reactions, injurious falls, quality of life, medical visits, emergency department visits, hospitalizations, and all-cause mortality. Random-effects meta-analyses were performed using the inverse variance method for similar studies, reporting risk ratios (RRs) or standardized mean differences (SMDs). Heterogeneity was assessed with *I*^2^ values, and publication bias was assessed with funnel plots and the Egger regression test.

**Results:**

Of the 14 649 records identified, 118 randomized clinical trials (comprising 417 412 patients) were included in this review. Interventions to address potentially inappropriate prescribing were associated with a reduction in the number of medications prescribed (SMD, −0.25 [95% CI, −0.38 to −0.13]), equivalent to approximately 0.5 fewer medications per patient. However, there were no substantial differences in the other outcomes, including nonserious adverse drug reactions (RR, 0.92 [95% CI, 0.58-1.46]), injurious falls (SMD, 0.01 [95% CI, −0.12 to 0.14]), quality of life (SMD, 0.09 [95% CI, −0.04 to 0.23]), medical visits (SMD, 0.02 [95% CI, −0.02 to 0.07]), emergency department admissions (RR, 1.02 [95% CI, 0.96-1.08]), hospitalizations (RR, 0.95 [95% CI, 0.89-1.02]), or all-cause mortality (RR, 0.94 [95% CI, 0.85-1.04]).

**Conclusions and Relevance:**

In this systematic review and meta-analysis, interventions to address potentially inappropriate prescribing were associated with reductions in the number of medications prescribed, with no substantial change in other outcomes. These findings suggest that inappropriate prescribing interventions may be implemented to safely reduce the number of medications prescribed to older adults in the primary care setting. Future studies should continue to evaluate these interventions using standardized criteria and consistently report potential harms to support data synthesis and capture key outcomes such as quality of life, hospitalization, and mortality.

## Key Points

**Question:**

Are interventions to reduce potentially inappropriate prescribing for older primary care patients associated with changes in prescribing, hospitalizations, and mortality?

**Findings:**

In this systematic review and meta-analysis of 118 randomized clinical trials, prescribing interventions targeting potentially inappropriate medications were safe and were associated with reductions in the number of medications prescribed. However, these interventions were not associated with substantial differences in hospitalizations and all-cause mortality.

**Meaning:**

These findings suggest that interventions to address potentially inappropriate prescribing can reduce the number of medications taken by older adults in primary care; however, their effects on other outcomes require further study.

## Teaser

This systematic review and meta-analysis evaluates the association between interventions to reduce potentially inappropriate prescribing for older primary care patients and changes in the number of medications prescribed, drug-related harms, hospitalizations, and mortality.

## Introduction

Potentially inappropriate prescribing (PIP), defined as prescribing medications with risks that may outweigh benefits or without a clinical indication,^1^ is common and associated with serious harms such as hospitalization and death.^2,3,4,5,6^ Moreover, patients prefer to take fewer medications for multiple reasons, including cost, convenience, and concerns about adverse effects.^7^ In willingness-to-pay studies, some patients reported they would be willing to pay substantial sums of money or even give up 12 weeks of life in order to stop taking 1 pill per day.^8,9^ The likelihood of PIP increases as a patient is prescribed more medications due in part to the risk of harmful drug-drug interactions, and older adults are more likely to be exposed to PIP.^5,10^

PIP can be countered by deprescribing, a shared decision-making process that involves stopping, reducing doses, or changing to alternative therapies.^11,12^ Interventions to address PIP are designed to curb the tendency to continue medications and to address several of the known barriers to deprescribing. For prescribers, such barriers include clinical practice guidelines that focus on one condition when patients may have several, a reluctance to discontinue a medication prescribed by another physician, time constraints, and gaps in information sharing between prescribers.^13^ Patients themselves are sometimes reluctant to discontinue medications due to unawareness that they should be stopped, fear of stopping a previously recommended treatment, challenges in communicating with prescribers, and uncertainty about how to appropriately discontinue a medication.^14,15,16^

Although PIP is associated with serious harms, previous studies have reported inconsistent effects of interventions on clinical outcomes.^11,17,18^ These earlier findings may reflect differences in the types of medications targeted, the design of interventions, or the relatively low risk associated with some deprescribed medications. Alternatively, the patients at highest risk of PIP may also be independently at the highest risk of adverse events related to their medical comorbidities and frailty. A clearer understanding of the effects of PIP interventions across settings, patient populations, and outcomes is needed to support evidence-based decision-making, and results of such studies will be used to inform clinical practice guidance for primary care clinicians.^19^ The purpose of this systematic review was to examine the association between interventions to address PIP in older adult outpatients and changes in outcomes reported in randomized clinical trials (RCTs).

## Methods

The protocol for this systematic review and meta-analysis was developed by the University of Ottawa Evidence Review and Synthesis Centre as part of the work of the Canadian Task Force on Preventive Health Care.^19^ The systematic review was registered with PROSPERO (CRD42022302313) and the Open Science Framework (URJ4B). The study followed the Preferred Reporting Items for Systematic Reviews and Meta-Analyses (PRISMA) reporting guideline.^20^ We will separately report results of a systematic review related to the acceptability of the interventions.

### Eligibility

We included RCTs of interventions to address PIPs in older adults (aged ≥65 years) residing in the community or in long-term care facilities such as nursing homes or assisted-living facilities. We included studies of any intervention aimed at reducing PIP, including reviews by health care practitioners who were either part of or independent from the clinical team. Eligible comparators included usual care or minimal interventions, such as generic reminders about safe prescribing. Studies were eligible regardless of whether the intervention addressed all medications or a specific class (eg, benzodiazepines, antipsychotics, or proton pump inhibitors), provided the aim was to reduce PIP.

The primary or critical outcomes were all-cause mortality, hospitalizations, and nonserious (ie, not requiring hospitalization and not causing death) adverse drug events. Secondary or important outcomes included quality of life, emergency department visits, medical visits, injurious falls, and number of medications. The outcomes were assigned as either critical or important as part of the Canadian Task Force on Preventive Health Care process for making recommendations.^21,22^

We included studies that reported any of the aforementioned outcomes. Studies were eligible if the population comprised primarily older adults (ie, the mean or median age was ≥65 years, or the majority of participants were aged ≥65). We excluded studies that solely or predominantly enrolled adults younger than 65 years and studies that enrolled only inpatients. We excluded studies that addressed only the prescribing of antibiotics, because antibiotic stewardship is a topic with its own literature.

### Information Sources and Search Strategy

An experienced medical information specialist (B.S.) developed the search strategy in consultation with the review team. The MEDLINE strategy was peer reviewed by another senior information specialist using the Peer Review of Electronic Search Strategies (PRESS) checklist.^23^ Using the OVID platform, the following databases were searched in multifile: MEDLINE, Embase Classic+Embase, and the Cochrane Central Register of Controlled Trials. Search terms included a combination of controlled vocabulary (eg, *polypharmacy*, *inappropriate prescribing*, and *health services for the aged*) and free text (eg, *deprescribing*, *excess drugs*, and *elderly*) (see the search strategy in eAppendix 1 in Supplement 1). An RCT filter was applied to the MEDLINE and Embase search strategies. There were no language or date restrictions but where possible, animal-only records and opinion pieces were removed from the results. The search was run from inception to September 6, 2024. We also searched websites for relevant studies (eAppendix 2 in Supplement 1).

### Study Selection

Following completion of a pilot exercise, titles and abstracts were screened by 2 independent reviewers (A.B., N.V., R.P., or F.A.) by applying the inclusion and exclusion criteria. Disagreements were resolved through discussion or by consulting with a third reviewer. Full-text articles were assessed against the inclusion and exclusion criteria by 2 independent reviewers (A.W., A.R., A.B., N.V., R.P., or F.A.), and disagreements were resolved through discussion.

### Data Abstraction and Risk of Bias Assessment

Three reviewers (N.P., A.W., and A.R.) developed and piloted a data abstraction tool that recorded information about the characteristics of each study, details about the intervention, and relevant outcomes. Two reviewers (A.W. and A.R.) extracted information about each study. We used the Cochrane revised risk of bias instrument for RCTs (also known as the RoB 2 tool) to assess all included studies.^24^

### Statistical Analysis

#### Synthesis of Included Studies

We tabulated the results by the type of intervention and the outcomes reported. We categorized interventions as either explicit (a review procedure applying rules that specifically mention medications to avoid) or implicit (a general review procedure without mention of specific medications).^19^ We statistically pooled results in a meta-analysis using a random-effects model applying the inverse variance method if 2 or more studies were similar in terms of participants and outcomes. Results are expressed as risk ratios (RRs) for dichotomous outcomes and as standardized mean differences (SMDs) for continuous outcomes. For cluster RCTs, we adjusted events and sample sizes based on the design effect that takes into account the cluster size and intracluster correlation.^25^ We assessed statistical heterogeneity using the restricted maximum likelihood inconsistency or *I*^2^ measure.^26^ To try to explain statistical heterogeneity, we performed subgroup analyses. For studies that could not be statistically pooled, we used the vote-counting method based on the direction of the effect.^27^ We assessed publication bias using funnel plots and the Egger regression test.^28^

#### Rating of Certainty and Drawing Conclusions

Two reviewers (A.W. and A.R.) rated the certainty of evidence for each outcome and agreed on the final rating and conclusion statement through discussion with a third reviewer (N.P.), where necessary. We used Grading of Recommendations Assessment, Development, and Evaluation (GRADE) guidance to assess certainty and draw conclusions by considering risk of bias, consistency of effect, imprecision, indirectness, and publication bias and to present findings in a summary of findings table (Table).^29^

**Table.** Summary of Findings for Interventions to Reduce PIP

Outcome	No. of trials	No. of patients	Group		Relative effect (95% CI)	Certainty of evidence	Conclusion
			PIP intervention	Usual care			
No. of medications, mean (SD)	43	16 174	9.4 (3.3)	9.9 (3.3)	SMD, 0.25 (−0.38 to −0.13)	High (heterogeneity noted)	Reduces
Rate of nonserious adverse reactions, No. (%)	3	841	213 (50.7)	282 (33.2)	RR, 0.92 (0.58-1.46)	Very low (imprecision, inconsistency)	May have little to no effect
No. of injurious falls per 100 patients, median (IQR)	21	10 963	3.5 (2.0-6.0)	3.2 (2.0-5.8)	SMD, 0.01 (−0.12 to 0.14)	Low (imprecision)	May result in little to no difference
Quality of life	37	12 221	Not calculable	Not calculable	SMD, 0.09 (−0.04 to 0.23)	Low (imprecision)	May result in little to no difference
No. of medical outpatient visits, mean (SD)	10	5341	5.6 (2.0)	5.5 (2.0)	SMD, 0.02 (−0.02 to 0.07)	Low (imprecision)	May result in little to no difference
Emergency department visits, No. (%)	11	5853	679 (23.2)	711 (24.3)	RR, 1.02 (0.96-1.08)	Low (imprecision)	May result in little to no difference
Hospitalizations, No. (%)	22	57 636	15 302 (53.1)	12 421 (43.1)	RR, 0.95 (0.89-1.02)	Low (imprecision)	May slightly reduce
Mortality, No. (%)	47	16 682	1101 (13.2)	1151 (13.8)	RR, 0.94 (0.85-1.04)	Moderate (imprecision)	Probably slightly reduces

Abbreviations: PIP, potentially inappropriate prescribing; RR, risk ratio; SMD, standardized mean difference.

Statistical significance was set at *P* < .05 (2-tailed). Meta-analysis was performed using Meta-Mar, version 3.5.1 (Meta-Mar).

## Results

### Search

**Figure 1.**
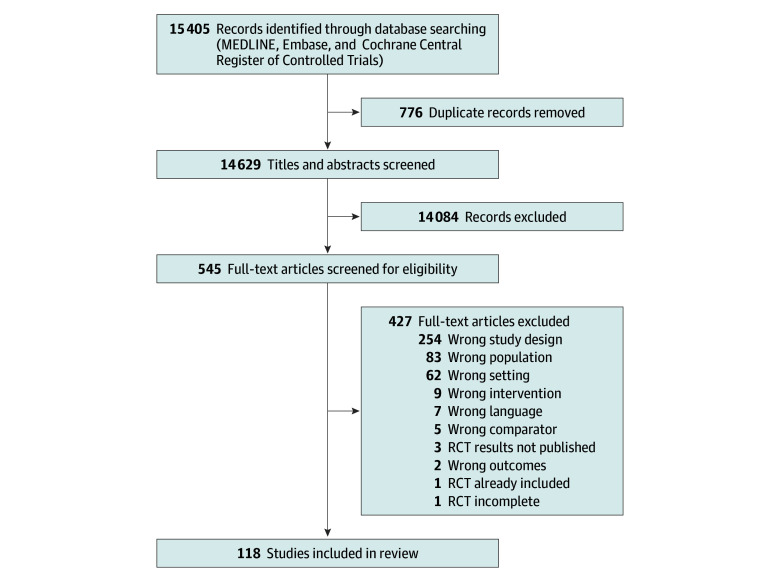
Study Flow Diagram RCT indicates randomized clinical trial.

After duplicates were removed, the search returned 14 629 unique records (Figure 1). We assessed full-text articles for 545 studies. We included 118 RCTs in this review,^11,30,31,32,33,34,35,36,37,38,39,40,41,42,43,44,45,46,47,48,49,50,51,52,53,54,55,56,57,58,59,60,61,62,63,64,65,66,67,68,69,70,71,72,73,74,75,76,77,78,79,80,81,82,83,84,85,86,87,88,89,90,91,92,93,94,95,96,97,98,99,100,101,102,103,104,105,106,107,108,109,110,111,112,113,114,115,116,117,118,119,120,121,122,123,124,125,126,127,128,129,130,131,132,133,134,135,136,137,138,139,140,141,142,143,144,145,146^ with a total of 417 412 patients (212 090 randomized to receive an intervention to address PIP and 205 322 randomized to the control). All included studies and their characteristics are listed in eTable 1 in Supplement 1. Examples of excluded studies with common reasons for exclusion are listed in eTable 2 in Supplement 1.

### Study Characteristics

Of the 118 included trials, 93 (79%) included outpatients in ambulatory care settings^30,31,33,34,37,38,39,40,41,43,44,45,46,47,48,49,50,51,52,53,54,56,57,58,59,60,61,62,63,65,66,67,69,70,71,72,74,75,76,79,82,83,84,85,86,87,88,89,90,91,92,93,95,96,100,101,102,103,104,105,106,107,109,110,111,112,113,115,116,117,118,119,120,123,126,127,128,129,131,132,133,134,135,138,139,140,141,143,145,146,147^ and 25 (21%) included long-term care facility residents.^32,35,36,42,55,68,73,77,78,80,81,94,97,98,99,108,114,121,122,124,125,130,136,137,142,144^ Most trials (68 [58%]) assessed implicit interventions,^30,31,32,33,37,38,39,40,43,48,49,52,54,56,57,58,59,60,62,65,66,67,68,70,71,72,74,75,76,78,79,80,82,84,85,86,87,89,90,91,93,95,98,99,101,102,104,106,110,111,113,114,116,117,119,121,124,127,129,130,133,134,135,137,142,143,145^ and the remainder (50 [42%]) assessed explicit interventions (eTable 1 in Supplement 1).^34,35,36,41,42,44,45,46,47,50,51,53,55,61,63,69,73,77,81,83,88,92,94,96,97,100,103,105,107,108,109,112,115,118,120,122,123,125,126,128,131,132,136,138,139,140,141,144,146,147^ The majority of interventions involved pharmacists (60 trials [51%]),^33,35,36,38,39,40,43,45,47,48,50,51,52,56,57,59,70,71,72,73,74,75,76,77,78,79,80,84,86,92,93,95,98,99,100,102,104,105,113,114,115,117,119,121,122,124,125,130,131,132,133,135,136,137,140,141,143,145,147^ and many (32 [27%]) involved software reviews of medication lists.^30,31,34,44,49,50,51,58,60,63,71,72,74,75,78,83,85,91,92,103,105,106,107,116,121,123,125,128,133,140,141,147^ The most common outcomes reported were the number of medications prescribed (47 trials [40%]),^31,33,37,40,42,43,44,51,52,54,56,62,65,66,67,69,73,75,76,77,80,84,85,87,88,92,93,95,96,99,100,101,102,103,104,106,107,110,112,114,116,117,118,120,125,126,129,136,137,141,142,143,145,146^ hospitalizations (43 [36%]),^31,36,38,40,45,46,48,52,55,62,69,76,81,82,85,86,87,88,89,91,100,101,102,106,111,112,114,116,117,118,121,123,124,125,126,133,134,135,136,142^ and all-cause mortality (53 [45%]).^31,36,39,40,46,48,51,52,55,69,71,72,73,75,76,78,80,84,85,88,89,91,93,94,95,96,98,99,101,103,105,112,115,116,117,118,119,120,121,123,124,125,126,127,129,135,136,137,140,141,143,147^ Most trials (80 [68%]) had a follow-up period between 6 and 12 months, with a range between 1.5 to 24 months.^30,31,32,33,34,35,36,37,38,39,40,41,42,43,44,45,46,47,48,49,50,51,52,53,54,55,56,57,58,59,60,61,62,63,65,66,67,68,69,70,71,72,73,74,75,76,77,78,79,80,81,82,83,84,85,86,87,88,89,90,91,92,93,94,95,96,97,98,99,100,101,102,103,104,105,106,107,108,109,110,111,112,113,114,115,116,117,118,119,120,121,122,123,124,125,126,127,128,129,130,131,132,133,134,135,136,137,138,139,140,141,142,143,144,145,146,147^

### Risk of Bias and Certainty

The risk of bias assessments identified 76 trials (64%) with low risk of bias,^30,32,33,34,36,38,39,40,44,45,46,47,48,49,50,53,54,55,56,57,59,60,61,63,69,70,71,72,73,74,77,78,79,80,82,83,84,85,87,90,91,93,94,95,96,97,98,100,102,105,109,110,111,112,114,115,119,120,121,123,124,126,128,129,130,131,133,135,136,137,139,140,141,145,146,148^ 40 (34%) with some risk of bias,^31,35,37,41,42,43,51,52,58,62,64,65,66,67,68,75,76,81,86,88,89,92,99,101,103,104,106,107,113,116,117,118,122,125,127,132,134,138,142,143^ and 2 (2%) with high risk of bias.^108,144^ Issues related to the randomization process, such as baseline differences, and missing outcome data were the most common reason for high risk of bias assessment (eFigures 1 and 2 in Supplement 1). In the meta-analysis results described next, we did not detect evidence of publication bias.

There were multiple RCTs reporting results for all outcomes. Our certainty ratings were downgraded due to imprecision for all outcomes except the number of medications (Table). For nonserious adverse reactions, results were not consistent in the 3 trials that reported them.^82,100,143^

### PIP Interventions by Outcome

**Figure 2.**
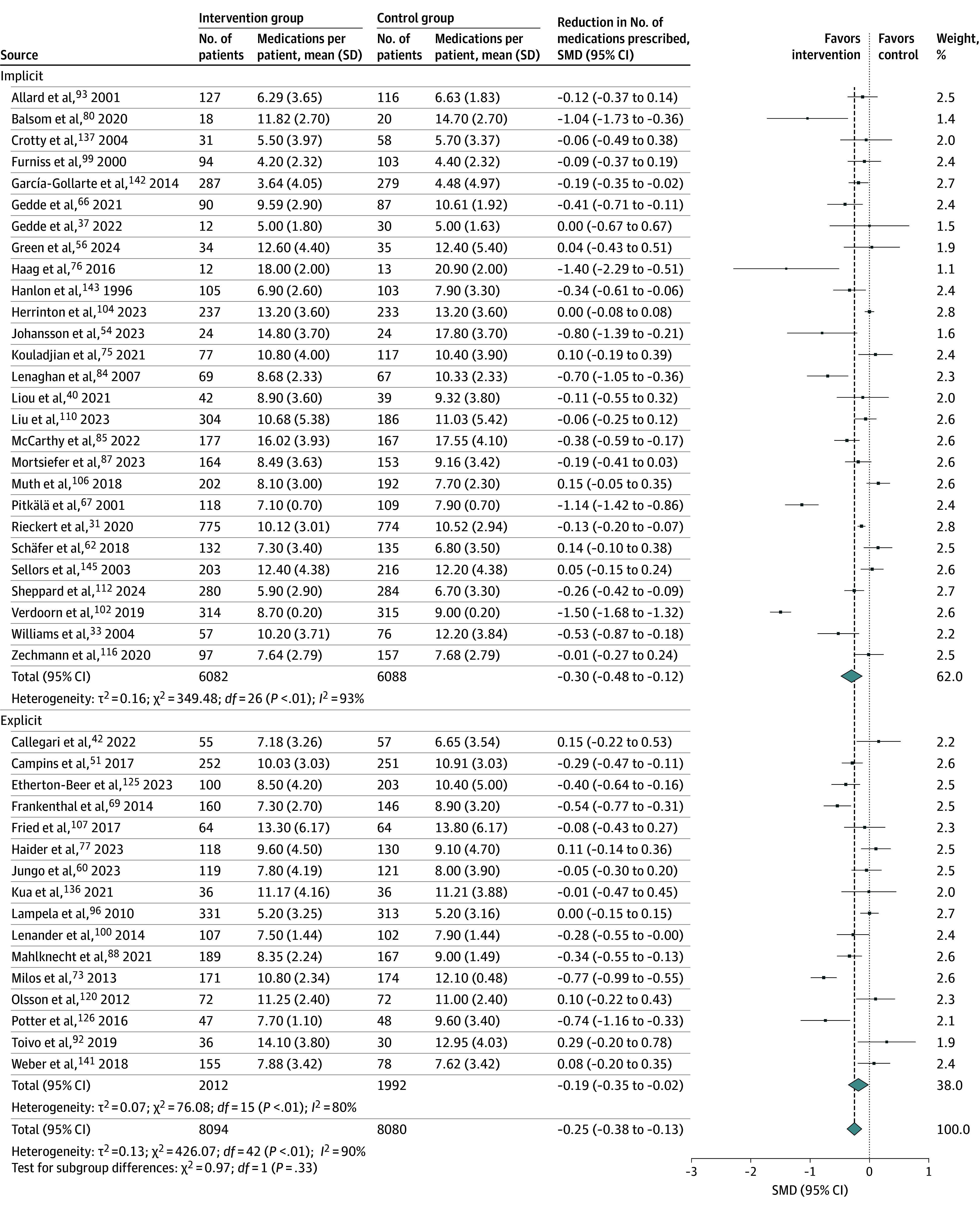
Changes in Number of Prescribed Medications Associated With Potentially Inappropriate Prescribing Interventions SMD indicates standardized mean difference.

The outcome of PIP interventions on the number of prescribed medications was reported in 47 trials,^31,33,37,40,42,43,51,52,54,56,60,62,64,66,67,69,73,75,76,77,80,84,85,87,88,92,93,96,99,100,102,104,106,107,110,112,116,120,125,126,129,136,137,141,142,143,145^ Of those, 43 trials could be pooled.^31,33,37,40,42,51,54,56,60,62,66,67,69,73,75,76,77,80,84,85,87,88,92,93,96,99,100,102,104,106,107,110,112,116,120,125,126,136,137,141,142,143,145^ PIP interventions were associated with a reduction in the number of medications prescribed (SMD, −0.25 [95% CI, −0.38 to −0.13]; *I*^2^ = 90%; n = 16 174) (Figure 2), which corresponds to a difference of approximately 0.5 medications per patient (the SD was 3.3 in control participants where patients were taking a mean of 9.9 medications at the end of trials). Trials not included in statistical pooling also reported reductions in the proportion of patients with prescriptions or a reduction in the mean number of prescriptions. The reduction in the number of medications was generally durable over the trial duration, but in 1 trial^42^ there was an initial effect after 3 months that was not sustained at 12 months. Many studies specifically reported on potentially inappropriate medications (PIMs), but variation in evaluation and reporting made statistical pooling difficult; of the 67 trials reporting on PIMs, 60 trials showed greater reductions in the intervention group,^30,32,33,34,35,39,40,44,46,47,48,50,51,53,54,55,58,59,60,61,63,65,66,68,69,70,73,76,77,81,82,83,85,86,90,92,93,94,95,96,97,103,105,107,109,110,113,115,118,122,124,127,128,129,130,137,138,140,143,144^ 6 showed greater reductions in the control group,^45,71,121,123,132,148^ and 1 showed no difference.^139^

Nonserious adverse reactions were reported in only 3 trials.^82,100,143^ There was no clear association between PIP interventions and the proportion of participants experiencing a nonserious adverse reaction (RR, 0.92 [95% CI, 0.58-1.46]; *I*^2^ = 0%; n = 841) (eFigure 3 in Supplement 1).

Injurious falls were reported in 24 trials. There was no association between PIP interventions and the number of injurious falls in the 21 trials reporting the rate of falls (SMD, 0.01 [95% CI, −0.12 to 0.14]; *I*^2^ = 80%; n = 10 963) (eFigure 4 in Supplement 1).^31,48,60,61,69,75,88,89,98,101,114,121,122,124,125,126,135,136,140,141,142^ In the 3 trials not statistically pooled, 2 trials^49,58^ reported fewer fallers in the intervention group and 1 trial^31^ reported more fallers in the intervention group.

Quality of life was reported in 37 trials.^31,40,42,43,46,51,54,59,60,62,69,70,72,82,84,85,88,89,94,101,102,105,106,108,111,113,116,118,119,120,121,122,125,126,139,143,146^ Most studies used the EuroQol 5-Dimension Questionnaire (27 [73%]).^40,43,46,51,54,60,62,72,82,84,85,88,89,101,102,106,108,113,116,119,120,121,122,125,126,139,146^ Other studies used the 36-Item Short Form Survey (4 [11%]),^31,59,143,146^ the 12-Item Short Form Survey (3 [8%]),^69,70,94^ or the Quality of Life in Late-Stage Dementia Scale (1 [3%]).^42^ There was no association between PIP interventions and quality of life (SMD, 0.09 [95% CI, −0.04 to 0.23]; *I*^2^ = 80%; n = 12 221) (eFigure 5 in Supplement 1). In the 2 trials not statistically pooled,^51,88^ both reported a reduction in quality-of-life scores in the intervention group.

Medical outpatient visits were reported in 18 trials.^40,51,52,57,62,75,82,85,86,100,102,104,117,118,122,126,142,147^ There was no association with the number of outpatient medical visits based on results from 10 trials reporting the number of visits (SMD, 0.02 [95% CI, −0.02 to 0.07]; *I*^2^ = 0%; n = 5341) (eFigure 6 in Supplement 1).^40,51,62,82,85,86,100,104,142,148^ In the 8 trials not statistically pooled, 2 reported fewer outpatients visits in the intervention group,^75,102^ 5 showed more visits,^57,117,118,122,126^ and 1 reported no difference.^52^

Emergency department visits were reported in 16 trials.^40,51,57,76,81,82,84,85,91,102,104,114,117,121,133,142^ There was no association with emergency department visits when data from 11 trials reporting the proportion of participants who visited the emergency department were pooled (RR, 1.02 [95% CI, 0.96-1.08]; *I*^2^ = 0%; n = 5853) (eFigure 7 in Supplement 1).^57,76,81,82,84,91,102,104,117,121,133^ In the 5 trials not statistically pooled, 2 trials reported fewer emergency department visits in the intervention group^51,142^ and 3 trials reported more.^40,85,114^

**Figure 3.**
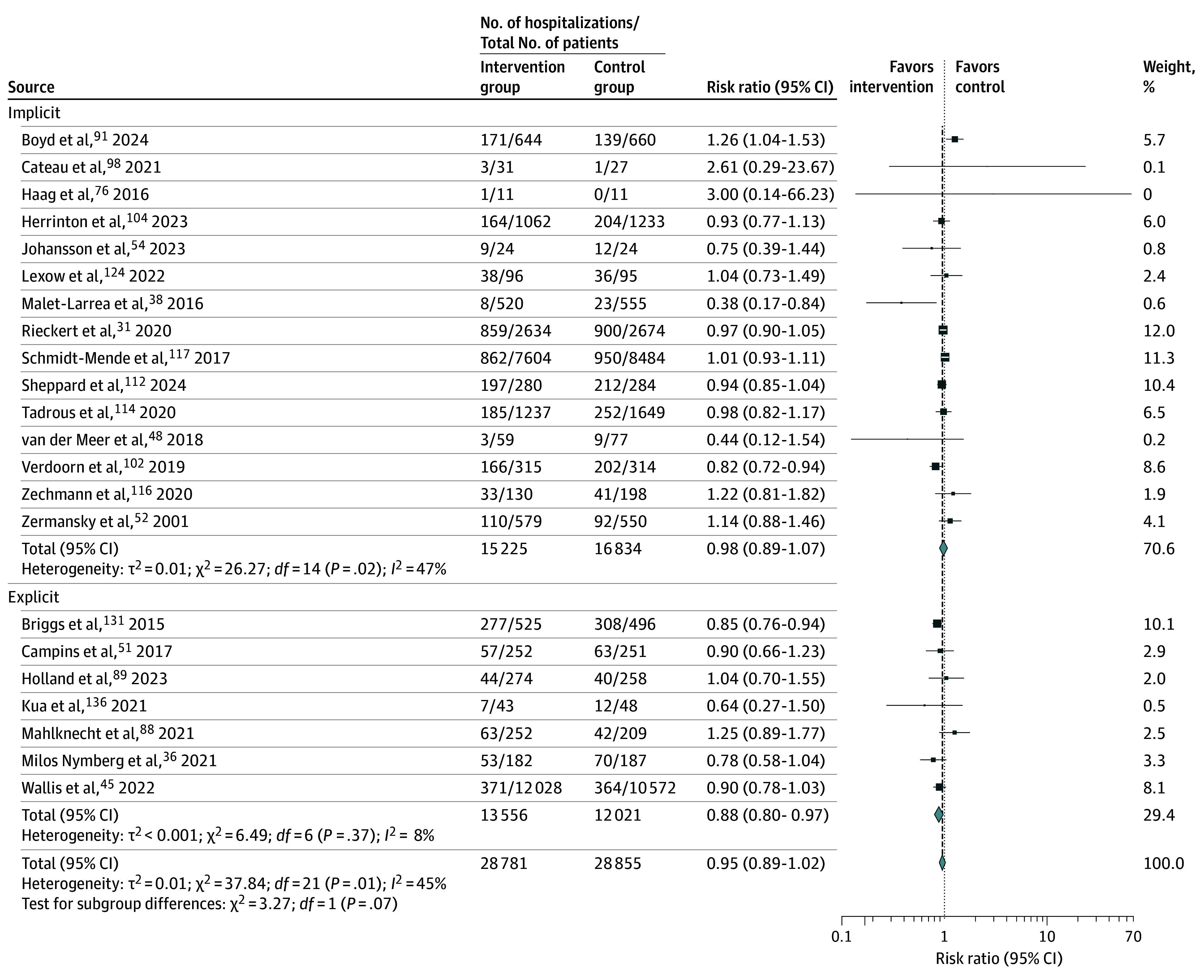
Changes in Hospitalizations Associated With Potentially Inappropriate Prescribing Interventions

Hospitalization rates were reported in 43 trials.^31,36,38,40,45,46,48,51,52,54,62,69,76,81,85,86,87,88,89,91,98,100,101,102,104,106,111,112,114,116,117,118,121,123,124,125,126,131,133,134,135,136,142^ There was a small reduction in hospitalizations that was not statistically significant when data from 22 trials reporting the proportion of patients admitted to hospital were pooled and meta-analyzed (RR, 0.95 [95% CI, 0.89-1.02]; *I*^2^ = 45%; n = 57 636) (Figure 3).^31,36,38,45,48,51,52,54,76,88,89,91,98,102,104,112,114,116,117,124,131,136^ There was a slight reduction in hospitalizations for explicit interventions (RR, 0.88 [95% CI, 0.80-0.97]; *I*^2^ = 8%; n = 25 577). In the 21 trials not statistically pooled, 11 reported fewer admissions in the intervention group, ^46,62,81,85,86,87,100,118,126,133,142^ 6 reported more,^40,69,106,111,121,135^ and 4 reported no effect.^101,123,125,134^

**Figure 4.**
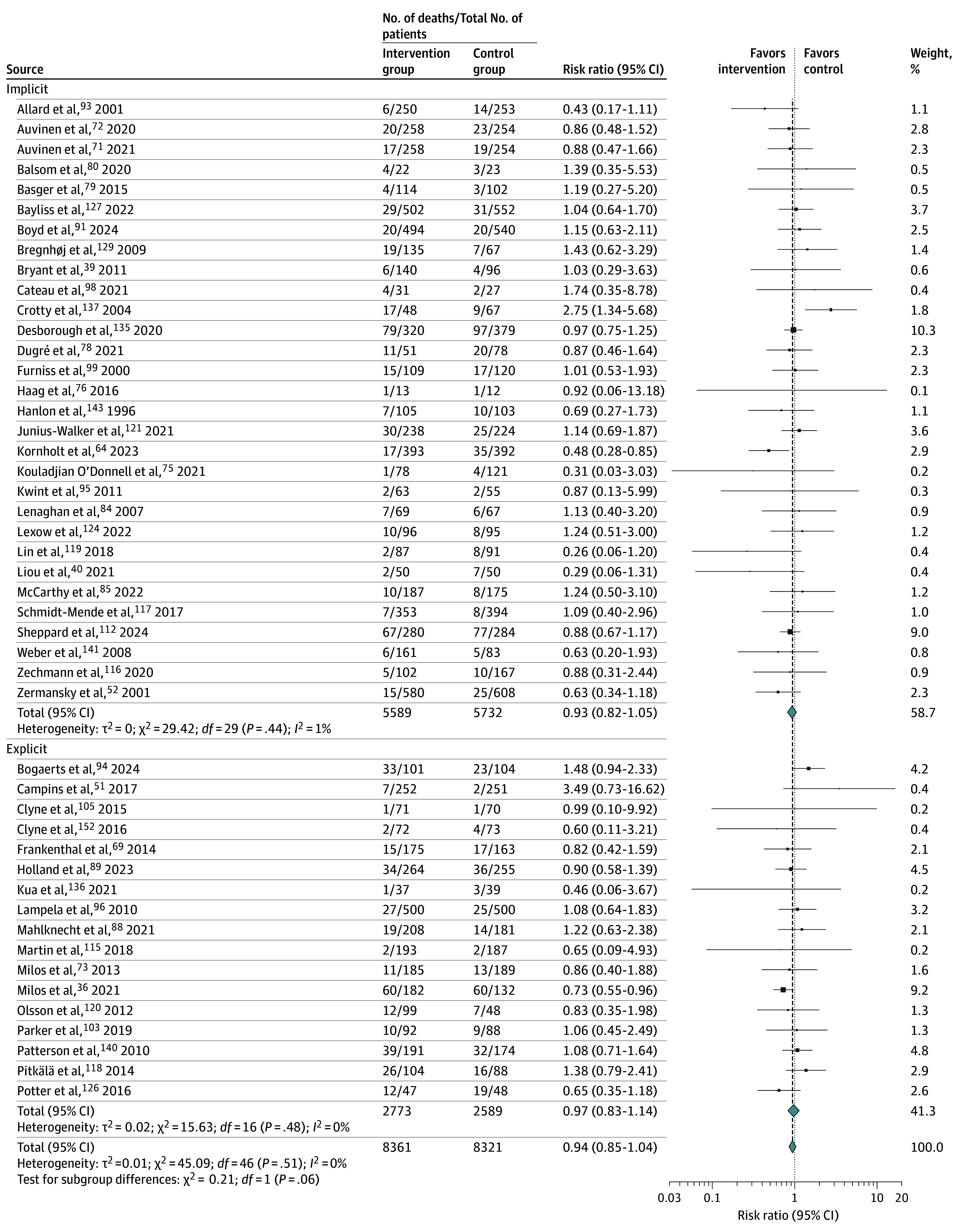
Changes in Mortality Associated With Potentially Inappropriate Prescribing Interventions

From the 53 articles that reported all-cause mortality, there was a slight reduction that was not statistically significant when data from 47 trials were pooled (RR, 0.94 [95% CI, 0.85-1.04]; *I*^2^ = 0%; n = 16 682) (Figure 4).^36,39,40,51,52,69,71,72,73,75,76,78,79,80,84,85,88,89,91,93,94,95,96,98,99,101,103,105,112,115,116,117,118,119,120,121,124,126,127,129,135,136,137,140,141,143,148^ In the 6 articles not pooled, 2 reported fewer deaths in the intervention group,^46,55^ 1 reported fewer deaths in the control group,^31^ and 3 reported no effect.^48,123,125^

### Sensitivity Analyses

Sensitivity analyses did not identify meaningful differences in outcomes based on patient characteristics (eg, baseline number of medications prescribed), intervention type (eg, explicit vs implicit interventions), intervention target (eg, PIMs only vs all medications), or RCT design (eg, duration of follow-up). None of these analyses substantially explained statistical heterogeneity. We also examined differences by care setting (ambulatory care vs long-term care facility or nursing home) and found no statistically significant interactions and overlapping CIs of effect estimates. Additionally, we conducted sensitivity analyses using different approaches to estimate the design effect for cluster trials, and these did not substantially change the results.

## Discussion

This systematic review of interventions to address PIP in older adult outpatients included 118 RCTs, with most assessed as having a low risk of bias. Meta-analysis showed that PIP interventions were associated with a reduction in the number of medications prescribed, without statistically significant differences in other outcomes such as hospitalizations and all-cause mortality. Although the CIs for some outcomes (eg, hospitalizations and mortality) were relatively wide, suggesting uncertainty, the point estimates from meta-analyses of a substantial number of trials suggest that PIP interventions may slightly reduce these outcomes. Outcomes such as outpatient medical visits had narrower CIs, indicating greater certainty around null effects.

For certain medications known to cause withdrawal symptoms (eg, sedative hypnotics, opioids, and proton pump inhibitors), it can be hypothesized that medical outpatient visits may be temporarily increased during tapering, based on our experience and consistent with the results of this review. Taken together, our results suggest that PIP interventions can likely be used to safely reduce the number of medications an older adult is prescribed in primary care. These findings may be reassuring to patients and to clinicians undertaking organized approaches to address PIP by deprescribing medications.

Our findings complement and expand on those of previous systematic reviews. However, our review included a wider range of interventions, including a variety of PIP interventions beyond deprescribing of specific drug classes, which likely contributed to the larger number of included studies. A 2024 umbrella review that included 12 systematic reviews across practice settings found that deprescribing was effective in reducing PIMs and in end-of-life care mortality but was not effective in reducing falls.^149^ A 2023 Cochrane systematic review of 38 studies, which included RCTs of interventions directed toward older adults, found uncertain effects on clinical outcomes but trends toward reduced PIMs.^150^ Approximately half of the included studies were conducted in hospital settings, in contrast to our review that focused on primary and long-term care settings.^17,150^ A 2023 systematic review of 32 RCTs, of which 15 were conducted in a hospital setting, found that deprescribing reduces PIMs and adverse drug reactions in older adults.^151^ Another review, limited to pharmaceutical interventions with PIM as a required outcome, identified only 14 studies, reflecting a more limited body of evidence when applying narrower inclusion criteria.^152^ A 2022 systematic review of interventional studies, conducted in mixed settings but predominantly hospitals, found that educational strategies appeared more effective in primary care settings.^148^ A 2016 systematic review of 12 trials in primary care found that based on a narrative summary, PIP interventions were effective at reducing PIP, but effects on clinical outcomes were uncertain.^147^ A 2024 updated systematic review of 259 studies found no substantial reduction in mortality overall, although subgroup analyses revealed significant reductions in mortality for participants aged 65 to 79 years (OR, 0.71 [95% CI, 0.51-0.99]; *P* = .04) and for patient-specific interventions (OR, 0.79 [95% CI, 0.63-0.99]; *P* = .04), supporting the safety and feasibility of deprescribing.^18^ A 2025 systematic review of emergency department–based programs similarly found limited evidence of benefit on clinical outcomes such as hospitalizations and adverse drug events, underscoring the ongoing uncertainty in this area.^153^ Previous knowledge syntheses have indicated that explicit interventions applied to specific patients seem to be more effective, whereas providing more general advice to clinicians does not seem to have any measurable benefit^154^; however, we found no difference between explicit and implicit interventions. Some studies have also observed reductions in the number of medications with no difference in the occurrence of adverse effects, potentially because some commonly stopped medications (eg, stool softeners or cholesterol-lowering medications) carry relatively small risks.^155^

### Limitations

This systematic review included a large number of clinical trials reporting a range of outcomes for multiple types of PIP interventions in older adults and can be used to help inform practice, but it has some important limitations. Although a variety of terms were used for the search, some studies could have been missed, because various terms are used to describe interventions addressing PIP. While most interventions were easy to classify as implicit or explicit, some were difficult to classify based on the reported descriptions. Different outcomes were reported in different trials, and outcomes such as adverse effects were reported in different ways and using different terms, sometimes limiting the ability to pool statistically. Not all trials reported mortality, and this outcome was ascertained differently across studies.^156^ There was also substantial statistical heterogeneity that was not readily explained by subgroup analyses for several outcomes, including hospitalization. Although the evidence was generally consistent across subgroups, some effects were influenced by a small number of large studies. For example, the observed reduction in hospitalizations in the explicit intervention subgroup was largely attributable to 2 trials and should be interpreted with this in mind. Effects could be different in different patient populations. Studies were conducted in different settings, with different types of supports available, so the findings may not be generalizable to every circumstance. There may be important benefits of reducing PIMs that were not directly assessed, such as reducing the burden of taking medications and reducing adverse effects that do not require care or are misattributed to aging or comorbidity.

## Conclusions

In this systematic review and meta-analysis of RCTs, PIP and the total number of medications were reduced in older adult outpatients without a substantial risk of harm. The outcomes of the interventions were uncertain, with an apparent slight reduction in hospitalizations and deaths. Future studies should continue to assess the effects of PIP interventions, and they should report negative effects of inappropriate medications using standardized criteria and terminology to facilitate the combining of future data and ensure the capture of important health outcomes, including quality of life, hospitalization, and mortality.

## Notes

Supplement 1. **eAppendix 1.** Search Strategy **eAppendix 2.** Gray Literature Sources **eTable 1.** Characteristics of Included Studies
eReferences
**eTable 2.** Examples of Excluded Studies **eFigure 1.** Summary of Risk of Bias Assessments for Included Studies **eFigure 2.** Risk of Bias Assessments for Each Included Trial **eFigure 3.** Nonserious Adverse Drug Reaction Effects of Potentially Inappropriate Prescribing Interventions **eFigure 4.** Falls Effects of Potentially Inappropriate Prescribing Interventions **eFigure 5.** Quality-of-Life Effects of Potentially Inappropriate Prescribing Interventions **eFigure 6.** Medical Visits Effects of Potentially Inappropriate Prescribing interventions **eFigure 7.** Emergency Department Visit Effects of Potentially Inappropriate Prescribing

Supplement 2.
Data Sharing Statement
