# Supplementary material for: Interventions to Address Potentially Inappropriate Prescribing for Older Primary Care Patients: A Systematic Review and Meta-Analysis
Source: JAMA Netw Open. 2025 Jun 27;8(6):e2517965. doi: 10.1001/jamanetworkopen.2025.17965 (PMC12205406; doi:10.1001/jamanetworkopen.2025.17965)
Supplement: Supplement 1. — eAppendix 1. Search Strategy eAppendix 2. Gray Literature Sources eTable 1. Characteristics of Included Studies eReferences eTable 2. Examples of Excluded Studies eFigure 1. Summary of Risk of Bias Assessments for Included Studies eFigure 2. Risk of Bias Assessments for Each Included Trial eFigure 3. Nonserious Adverse Drug Reaction Effects of Potentially Inappropriate Prescribing Interventions eFigure 4. Falls Effects of Potentially Inappropriate Prescribing Interventions eFigure 5. Quality-of-Life Effects of Potentially Inappropriate Prescribing Interventions eFigure 6. Medical Visits Effects of Potentially Inappropriate Prescribing interventions eFigure 7. Emergency Department Visit Effects of Potentially Inappropriate Prescribing [file jamanetwopen-e2517965-s001.pdf]

Supplemental Online Content

Persaud N, Workentin A, Rizvi A, et al. Interventions to address potentially inappropriate prescribing for older primary care patients: a systematic review and meta-analysis. *JAMA Netw Open*. 2025;8(6):e2517965. doi:10.1001/jamanetworkopen.2025.17965

eAppendix 1. Search Strategy

eAppendix 2. Gray Literature Sources

eTable 1. Characteristics of Included Studies

eReferences

eTable 2. Examples of Excluded Studies

eFigure 1. Summary of Risk of Bias Assessments for Included Studies

eFigure 2. Risk of Bias Assessments for Each Included Trial

eFigure 3. Nonserious Adverse Drug Reaction Effects of Potentially Inappropriate Prescribing Interventions

eFigure 4. Falls Effects of Potentially Inappropriate Prescribing Interventions

eFigure 5. Quality-of-Life Effects of Potentially Inappropriate Prescribing Interventions

eFigure 6. Medical Visits Effects of Potentially Inappropriate Prescribing interventions

eFigure 7. Emergency Department Visit Effects of Potentially Inappropriate Prescribing

This supplemental material has been provided by the authors to give readers additional information about their work.

© 2025 Persaud N et al. *JAMA Network Open*.

eAppendix 1. Search Strategy

Database**: Embase Classic+Embase** <1947 to 2024 September 05>, **Ovid MEDLINE(R)** <1946 to September 05, 2024>, **EBM Reviews - Cochrane Central Register of Controlled Trials** <Sept 2024>

---

1 exp Polypharmacy/ (33785)
2 (polypharm* or poly-pharm*).tw,kw,kf. (38323)
3 (polymedic* or poly-medic*).tw,kw,kf. (2273)
4 (polyprescri* or poly-prescri*).tw,kw,kf. (32)
5 (polypragmas* or poly-pragmas*).tw,kw,kf. (340)
6 Deprescriptions/ (3459)
7 (deprescri* or de-prescri*).tw,kw,kf. (6547)
8 ((cancel* or ceas* or cessation? or discontinu* or halt* or stop* or terminat*) adj2 (medicat* or medicine? or prescrib* or prescription?)).tw,kw,kf. (23221)
9 ((cancel* or ceas* or cessation? or discontinu* or halt* or stop* or terminat*) adj (drug or drugs)).tw,kw,kf. (2148)
10 Inappropriate Prescribing/ (10004)
11 ((appropriate* or inappropriate* or incorrect* or indiscriminat* or unnecessar* or rational* or irrational* or optimal* or optimum or suboptim* or sub-optim*) adj2 (medicat* or medicine? or prescrib* or prescription? or OTC or "over-the-counter" or "behind-the-counter")).tw,kw,kf. (48148)
12 ((appropriate* or inappropriate* or incorrect* or indiscriminat* or unnecessar* or rational* or irrational* or optimal* or optimum or suboptim* or sub-optim*) adj (drug or drugs)).tw,kw,kf. (26095)
13 ((excess* or multipl* or "five or more" or "5 or more") adj2 (medicat* or medicine? or prescrib* or coprescrib* or co-prescrib* or prescription? or coprescription? or co-prescription? or OTC or "over-the-counter")).tw,kw,kf. (16708)
14 ((excess* or multipl* or "five or more" or "5 or more") adj (drug or drugs)).tw,kw,kf. (27213)
15 (many medication* or many medicine* or many drug?).tw,kw,kf. (19012)
16 ((concomitant* or concurrent*) adj2 (medicat* or medicine? or prescrib* or prescription? or OTC or "over-the-counter" or "behind-the-counter")).tw,kw,kf. (21298)
17 ((concomitant* or concurrent*) adj (drug or drugs)).tw,kw,kf. (5008)
18 ((omit* or omission?) adj2 (medicat* or medicine? or prescrib* or prescription? or drug or drugs)).tw,kw,kf. (2191)
19 ((under$2 or over$2) adj2 (medicat* or prescrib* or prescription?)).tw,kw,kf. (22628)
20 (underprescri* or overprescri*).tw,kw,kf. (4353)
21 (quality adj2 (prescrib* or prescription?)).tw,kw,kf. (3111)
22 ((multidrug? or multi-drug?) adj2 (pharmacotherap* or pharmaco-therap* or prescrib* or prescription? or regim* or therap* or treatment?)).tw,kw,kf. (15659)
23 (multiple adj (pharmacotherap* or pharmaco-therap*)).tw,kw,kf. (108)
24 ((medication? or medicine? or prescrib* or prescription?) adj3 cascad*).tw,kw,kf. (499)
25 ((medication? or medicine? or prescrib* or prescription?) adj3 continuum*).tw,kw,kf. (223)
26 ((medication? or medicine? or prescrib* or prescription?) adj3 legac*).tw,kw,kf. (164)
27 ((medication? or medicine? or prescrib* or prescription?) adj3 (multi* chronic* or multi* comorbid* or multi* co-morbid* or multimorbid* or multi-morbid*)).tw,kw,kf. (911)
28 (prescrib* adj3 (harm or harmed or harms or harming or harmful*)).tw,kw,kf. (525)
29 Potentially Inappropriate Medication List/ (4095)
30 STOPP.tw,kw,kf. (2058)
31 "STOPP/START".tw,kw,kf. (977)
32 ((Beers or McLeod or NPS) adj2 criteri*).tw,kw,kf. (3016)
33 "Fit fOR The Aged".tw,kw,kf. (382)
34 ((FORTA or RASP or Pricus) adj2 (criteri* or instrument? or list*)).tw,kw,kf. (117)
35 Assessing Care of Vulnerable Elderly.tw,kw,kf. (2)
36 ACOVE.tw,kw,kf. (194)
37 Medication Appropriateness Index.tw,kw,kf. (447)
38 Medication Regimen Complexity.tw,kw,kf. (669)
39 Prescribing Optimi#ation Method.tw,kw,kf. (5)

© 2025 Persaud N et al. *JAMA Network Open*.

40 Systematic Tool to Reduce Inappropriate Prescribing.tw,kw,kf. (28)
41 (strip adj5 (medication? or medicine? or prescrib* or prescription?)).tw,kw,kf. (88)
42 or/1-41 [POLYPHARMACY] (270591)
43 Adult/ (15850941)
44 exp Aged/ (7679822)
45 aged.ti,kw,kf. (211932)
46 ((age? or year?) adj2 ("65" or "66" or "67" or "68" or "69" or "70" or "71" or "72" or "73" or "74" or "75" or "76" or "77" or "78" or "79" or "80" or "81" or "82" or "83" or "84" or "85" or "86" or "87" or "88" or "89" or "90" or "91" or "92" or "93" or "94" or "95" or "96" or "97" or "98" or "99" or "100")).tw,kw,kf. (2509883)
47 (elder? or elderly or geriatric* or gerontolog* or old-age? or senior?).tw,kw,kf. (1139002)
48 (older adj2 (adult* or female? or male? or man or men or patient? or person? or people? or population? or wom#n)).tw,kw,kf. (742600)
49 Retirement/ (31745)
50 (retiree? or retired or retirement).tw,kw,kf. (64527)
51 (boomer? or babyboomer* or baby-boomer*).tw,kw,kf. (4118)
52 Health Services for the Aged/ (56948)
53 Homes for the Aged/ (28173)
54 ((old age or nursing or longterm care or long-term care or LTC) adj (facilit* or home? or residen*)).tw,kw,kf. (118973)
55 or/43-54 [SENIOR FILTER] (20114408)
56 42 and 55 [POLYPHARMACY - SENIOR FILTER] (126788)
57 exp Animals/ not Humans/ (17886400)
58 56 not 57 [ANIMAL-ONLY REMOVED] (107542)
59 (comment or editorial or news or newspaper article).pt. (2581389)
60 (letter not (letter and randomized controlled trial)).pt. (2605095)
61 58 not (59 or 60) [OPINION PIECES REMOVED] (105716)
62 (controlled clinical trial or randomized controlled trial or pragmatic clinical trial or equivalence trial).pt. (713189)
63 Clinical Trials as Topic/ (307468)
64 exp Randomized Controlled Trials as Topic/ (459293)
65 (randomi#ed or randomi#ation? or randomly or RCT or placebo*).tw,kw,kf. (3186611)
66 ((singl* or doubl* or trebl* or tripl*) adj (mask* or blind* or dumm*)).tw,kw,kf. (509854)
67 trial.ti. (762148)
68 or/62-67 [RCT FILTER] (3999845)
69 61 and 68 [POLYPHARMACY - SENIORS - RCTs] (13504)
70 (202204* or 202205* or 202206* or 202207* or 202208* or 202209* or 202210* or 202211* or 202212* or 2023* or 2024*).dt. (3861460)
71 69 and 70 [UPDATE PERIOD] (573)
72 71 use medall [MEDLINE RECORDS] (573)
73 exp polypharmacy/ (33785)
74 (polypharm* or poly-pharm*).tw,kw,kf. (38323)
75 (polymedic* or poly-medic*).tw,kw,kf. (2273)
76 (polyprescri* or poly-prescri*).tw,kw,kf. (32)
77 (polypragmas* or poly-pragmas*).tw,kw,kf. (340)
78 deprescription/ (3459)
79 (deprescri* or de-prescri*).tw,kw,kf. (6547)
80 ((cancel* or ceas* or cessation? or discontinu* or halt* or stop* or terminat*) adj2 (medicat* or medicine? or prescrib* or prescription?)).tw,kw,kf. (23221)
81 ((cancel* or ceas* or cessation? or discontinu* or halt* or stop* or terminat*) adj (drug or drugs)).tw,kw,kf. (2148)
82 exp prescribing error/ (5190)
83 ((appropriate* or inappropriate* or incorrect* or indiscriminat* or unnecessar* or rational* or irrational* or optimal* or optimum or suboptim* or sub-optim*) adj2 (medicat* or medicine? or prescrib* or prescription? or OTC or "over-the-counter" or "behind-the-counter")).tw,kw,kf. (48148)
84 ((appropriate* or inappropriate* or incorrect* or indiscriminat* or unnecessar* or rational* or irrational* or optimal* or optimum or suboptim* or sub-optim*) adj (drug or drugs)).tw,kw,kf. (26095)

© 2025 Persaud N et al. *JAMA Network Open*.

85 ((excess* or multipl* or "five or more" or "5 or more") adj2 (medicat* or medicine? or prescrib* or coprescrib* or co-prescrib* or prescription? or coprescription? or co-prescription? or OTC or "over-the-counter")).tw,kw,kf. (16708)
86 ((excess* or multipl* or "five or more" or "5 or more") adj (drug or drugs)).tw,kw,kf. (27213)
87 (many medication* or many medicine* or many drug?).tw,kw,kf. (19012)
88 ((concomitant* or concurrent*) adj2 (medicat* or medicine? or prescrib* or prescription? or OTC or "over-the-counter" or "behind-the-counter")).tw,kw,kf. (21298)
89 ((concomitant* or concurrent*) adj (drug or drugs)).tw,kw,kf. (5008)
90 ((omit* or omission?) adj2 (medicat* or medicine? or prescrib* or prescription? or drug or drugs)).tw,kw,kf. (2191)
91 ((under$2 or over$2) adj2 (medicat* or prescrib* or prescription?)).tw,kw,kf. (22628)
92 (underprescri* or overprescri*).tw,kw,kf. (4353)
93 (quality adj2 (prescrib* or prescription?)).tw,kw,kf. (3111)
94 ((multidrug? or multi-drug?) adj2 (pharmacotherap* or pharmaco-therap* or prescrib* or prescription? or regim* or therap* or treatment?)).tw,kw,kf. (15659)
95 (multiple adj (pharmacotherap* or pharmaco-therap*)).tw,kw,kf. (108)
96 ((medication? or medicine? or prescrib* or prescription?) adj3 cascad*).tw,kw,kf. (499)
97 ((medication? or medicine? or prescrib* or prescription?) adj3 continuum*).tw,kw,kf. (223)
98 ((medication? or medicine? or prescrib* or prescription?) adj3 legac*).tw,kw,kf. (164)
99 ((medication? or medicine? or prescrib* or prescription?) adj3 (multi* chronic* or multi* comorbid* or multi* co-morbid* or multimorbid* or multi-morbid*)).tw,kw,kf. (911)
100 (prescrib* adj3 (harm or harmed or harms or harming or harmful*)).tw,kw,kf. (525)
101 potentially inappropriate medication/ (4295)
102 STOPP.tw,kw,kf. (2058)
103 "STOPP/START".tw,kw,kf. (977)
104 ((Beers or McLeod or NPS) adj2 criteri*).tw,kw,kf. (3016)
105 "Fit fOR The Aged".tw,kw,kf. (382)
106 ((FORTA or RASP or Pricus) adj2 (criteri* or instrument? or list*)).tw,kw,kf. (117)
107 Assessing Care of Vulnerable Elderly.tw,kw,kf. (2)
108 ACOVE.tw,kw,kf. (194)
109 Medication Appropriateness Index.tw,kw,kf. (447)
110 Medication Regimen Complexity.tw,kw,kf. (669)
111 Prescribing Optimi#ation Method.tw,kw,kf. (5)
112 Systematic Tool to Reduce Inappropriate Prescribing.tw,kw,kf. (28)
113 (strip adj5 (medication? or medicine? or prescrib* or prescription?)).tw,kw,kf. (88)
114 or/73-113 [POLYPHARMACY] (267713)
115 adult/ (15850941)
116 exp aged/ (7679822)
117 aged.ti,kw,kf. (211932)
118 ((age? or year?) adj2 ("65" or "66" or "67" or "68" or "69" or "70" or "71" or "72" or "73" or "74" or "75" or "76" or "77" or "78" or "79" or "80" or "81" or "82" or "83" or "84" or "85" or "86" or "87" or "88" or "89" or "90" or "91" or "92" or "93" or "94" or "95" or "96" or "97" or "98" or "99" or "100")).tw,kw,kf. (2509883)
119 (elder? or elderly or geriatric* or gerontolog* or old-age? or senior?).tw,kw,kf. (1139002)
120 (older adj2 (adult* or female? or male? or man or men or patient? or person? or people? or population? or wom#n)).tw,kw,kf. (742600)
121 retirement/ (31745)
122 (retiree? or retired or retirement).tw,kw,kf. (64527)
123 (boomer? or babyboomer* or baby-boomer*).tw,kw,kf. (4118)
124 elderly care/ (43152)
125 exp geriatric care/ (29317)
126 home for the aged/ (13801)
127 ((old age or nursing or longterm care or long-term care or LTC) adj (facilit* or home? or residen*)).tw,kw,kf. (118973)
128 or/115-127 [SENIOR FILTER] (20116432)
129 114 and 128 [POLYPHARMACY - SENIOR FILTER] (125796)

© 2025 Persaud N et al. *JAMA Network Open*.

130 (exp animal/ or exp animal model/ or exp animal experiment/ or nonhuman/ or exp vertebrate/) not (exp human/ or exp human experiment/) (13581592)
131 129 not 130 [ANIMAL-ONLY REMOVED] (125113)
132 editorial.pt. (1526661)
133 letter.pt. not (letter.pt. and randomized controlled trial/) (2598413)
134 131 not (132 or 133) [OPINION PIECES REMOVED] (123253)
135 exp randomized controlled trial/ or controlled clinical trial/ (1756489)
136 clinical trial/ (1652166)
137 exp "controlled clinical trial (topic)"/ (291143)
138 (randomi#ed or randomi#ation? or randomly or RCT or placebo*).tw,kw,kf. (3186611)
139 ((singl* or doubl* or trebl* or tripl*) adj (mask* or blind* or dumm*)).tw,kw,kf. (509854)
140 trial.ti. (762148)
141 or/135-140 (4748933)
142 134 and 141 [POLYPHARMACY - SENIORS - RCTs] (19765)
143 conference abstract.pt. (5233610)
144 142 not 143 (14949)
145 142 and 143 (4816)
146 limit 145 to yr="2022-current" (845)
147 144 or 146 [MOST RECENT 2 YEARS CONFERENCE ABSTRACTS RETAINED] (15794)
148 (202204* or 202205* or 202206* or 202207* or 202208* or 202209* or 202210* or 202211* or 202212* or 2023* or 2024*).dc. (5189138)
149 147 and 148 [UPDATE PERIOD] (2306)
150 149 use emczd [EMBASE RECORDS] (2306)
151 72 or 150 [BOTH DATABASES] (2879)
152 remove duplicates from 151 (2379) [TOTAL UNIQUE RECORDS – UPDATE PERIOD]
153 152 use medall [MEDLINE UNIQUE RECORDS - UPDATE PERIOD] (572)
154 152 use emczd [EMBASE UNIQUE RECORDS - UPDATE PERIOD] (1807)

****
Cochrane Library

Date Run: 06/09/2024 23:06:05

ID Search Hits
#1 [mh Polypharmacy] 411
#2 (polypharm* or poly-pharm*):ti,ab,kw 1574
#3 (polymedic* or poly-medic*):ti,ab,kw 108
#4 (polyprescri* or poly-prescri*):ti,ab,kw 2
#5 (polypragmas* or poly-pragmas*):ti,ab,kw 5
#6 [mh Deprescriptions] 115
#7 (deprescri* or de-prescri*):ti,ab,kw 415
#8 ((cancel* or ceas* or cessation* or discontinu* or halt* or stop* or terminat*) NEAR/2 (medicat* or medicine* or prescrib* or prescription*)):ti,ab,kw 2866
#9 ((cancel* or ceas* or cessation* or discontinu* or halt* or stop* or terminat*) NEXT (drug or drugs)):ti,ab,kw 242
#10 [mh "Inappropriate Prescribing"] 288
#11 ((appropriate* or inappropriate* or incorrect* or indiscriminat* or unnecessar* or rational* or irrational* or optimal* or optimum or suboptim* or sub-optim*) NEAR/2 (medicat* or medicine* or prescrib* or prescription* or OTC or "over-the-counter" or "behind-the-counter")):ti,ab,kw 2544
#12 ((appropriate* or inappropriate* or incorrect* or indiscriminat* or unnecessar* or rational* or irrational* or optimal* or optimum or suboptim* or sub-optim*) NEXT (drug or drugs)):ti,ab,kw 1530
#13 ((excess* or multipl* or "five or more" or "5 or more") NEAR/2 (medicat* or medicine* or prescrib* or coprescrib* or co-prescrib* or prescription* or coprescription* or co-prescription* or OTC or "over-the-counter")):ti,ab,kw 748
#14 ((excess* or multipl* or "five or more" or "5 or more") NEXT (drug or drugs)):ti,ab,kw 2514
#15 (many NEXT (medication* or medicine* or drug or drugs)):ti,ab,kw 477

#16 ((concomitant* or concurrent*) NEAR/2 (medicat* or medicine* or prescrib* or prescription* or OTC or "over-the-counter" or "behind-the-counter")):ti,ab,kw 3693
#17 ((concomitant* or concurrent*) NEXT (drug or drugs)):ti,ab,kw 314
#18 ((omit* or omission*) NEAR/2 (medicat* or medicine* or prescrib* or prescription* or drug or drugs)):ti,ab,kw 103
#19 ((under or over or overly) NEAR/2 (medicat* or prescrib* or prescription*)):ti,ab,kw 1983
#20 (underprescri* or overprescri*):ti,ab,kw 255
#21 (quality NEAR/2 (prescrib* or prescription*)):ti,ab,kw 495
#22 ((multidrug* or multi-drug*) NEAR/2 (pharmacotherap* or pharmaco-therap* or prescrib* or prescription* or regim* or therap* or treatment*)):ti,ab,kw 819
#23 (multiple NEXT (pharmacotherap* or pharmaco-therap*)):ti,ab,kw 7
#24 ((medication* or medicine* or prescrib* or prescription?) NEAR/3 cascad*):ti,ab,kw 6
#25 ((medication* or medicine* or prescrib* or prescription?) NEAR/3 continuum*):ti,ab,kw 2
#26 ((medication* or medicine* or prescrib* or prescription?) NEAR/3 legac*):ti,ab,kw 0
#27 ((medication* or medicine* or prescrib* or prescription?) NEAR/3 (multi* chronic or multi* comorbid* or multi* co-morbid* or multimorbid* or multi-morbid*)):ti,ab,kw 5100
#28 (prescrib* NEAR/3 (harm or harmed or harms or harming or harmful*)):ti,ab,kw36
#29 [mh "Potentially Inappropriate Medication List"] 75
#30 STOPP:ti,ab,kw 172
#31 "STOPP/START":ti,ab,kw75
#32 ((Beers or McLeod or NPS) NEAR/2 criteri*):ti,ab,kw 74
#33 "Fit fOR The Aged":ti,ab,kw 5
#34 ((FORTA or RASP or Pricus) NEAR/2 (criteri* or instrument* or list*)):ti,ab,kw 22
#35 "Assessing Care of Vulnerable Elderly":ti,ab,kw 0
#36 ACOVE:ti,ab,kw 12
#37 "Medication Appropriateness Index":ti,ab,kw 79
#38 "Medication Regimen Complexity":ti,ab,kw 31
#39 ("Prescribing Optimisation Method" or "Prescribing Optimization Method"):ti,ab,kw 2
#40 "Systematic Tool to Reduce Inappropriate Prescribing":ti,ab,kw 6
#41 (strip NEAR/5 (medication* or medicine* or prescrib* or prescription*)):ti,ab,kw 13
#42 {or #1-#41} 22832
#43 [mh ^Adult] 439524
#44 [mh Aged] 278605
#45 aged:ti,kw 548960
#46 ((age or aged or ages or year or years) NEAR/2 ("65" or "66" or "67" or "68" or "69" or "70" or "71" or "72" or "73" or "74" or "75" or "76" or "77" or "78" or "79" or "80" or "81" or "82" or "83" or "84" or "85" or "86" or "87" or "88" or "89" or "90" or "91" or "92" or "93" or "94" or "95" or "96" or "97" or "98" or "99" or "100")):ti,ab,kw 254020
#47 (elder or elders or elderly or geriatric* or gerontolog* or "old-age" or "old-aged" or senior or seniors):ti,ab,kw 138087
#48 (older NEAR/2 (adult* or female or females or male or males or man or men or patient or patients or person or persons or people or peoples or population* or woman or women)):ti,ab,kw 42101
#49 [mh Retirement] 101
#50 (retiree* or retired or retirement):ti,ab,kw 1074
#51 (boomer* or babyboomer* or baby-boomer*):ti,ab,kw 84
#52 [mh "Health Services for the Aged"] 580
#53 [mh "Homes for the Aged"] 828
#54 (("old age" or nursing or "longterm care" or "long-term care" or LTC) NEXT (facilit* or home or homes or residen*)):ti,ab,kw 6372
#55 {or #43-#54} 866858
#56 #42 and #55 10830
#57 conference proceeding:pt 247486
#58 #56 NOT #57 9278
#59 #56 AND #57 with Publication Year from 2022 to 2024, in Trials 269
#60 #58 OR #59 9547
#61 #58 OR #59 with Cochrane Library publication date Between Mar 2022 and Dec 2024, in Trials 1684

© 2025 Persaud N et al. *JAMA Network Open*.

eAppendix 2. Gray Literature Sources

Examples of websites searched

https://deprescribingresearch.org/

https://deprescribing.org/

https://deprescribing.eu/

https://www.australiandeprescribingnetwork.com.au/

https://www.nice.org.uk/

https://opentrials.net/

https://clinicaltrials.gov/

https://www.who.int/clinical-trials-registry-platform

© 2025 Persaud N et al. *JAMA Network Open*.

eTable 1. Characteristics of Included Studies

Study	Trial design	Setting and population	Intervention type and description	Outcomes	Follow up duration
1. Allard et al 2001	Randomized controlled trial	Outpatient primary care patients > 75 years old, in Quebec, Canada	Implicit.<br>Medication review by GP, pharmacist and nurse using the following criteria: indication, effectiveness, dosage, instructions and their applicability, drug interactions, drug–pathology interactions, therapeutic overlapping, duration of treatment and cost	Mortality, Number of medications, PIMs	12 months
2. Almutairi et al 2023	Cluster randomized controlled trial at facility level	Residential aged care facility patients ≥ 65 years old, in Western Australia	Explicit.<br>Dementia Training Australia's Medication Management Consultancy provided online education, medication audits, resources for optimizing psychotropic drug use, non-pharmacological deprescribing strategies, and staff education on behavioral and psychological symptoms of dementia	Hospitalizations, ED visits, PIMs	12 months
3. Amorim et al 2024	Randomized controlled trial	General practitioners working at the primary care facilities in Vitoria de Conquista, Brazil	Implicit.<br>Mobile application called MPI Brazil provided which aimed to help reduce inappropriate prescribing in older adults by offering PIP info, therapeutic alternatives, and deprescribing guidelines. GPs used it alongside the MedSUS app for medication info on Android tablets during consultations for at least three months.	PIMs	3 months
4. Auvinen et al 2020	Randomized controlled trial	Public home care patients ≥ 65 years old in five areas in Finland: Forssa, Haapajärvi, Lahti, Juva and Savonlinna	Implicit.<br>Medication review by GP, pharmacist and nurse using SFINX, RENBASE, PHARAO, and Meds75 databases	Mortality, Quality of life	6 months
5. Auvinen et al 2021	Randomized controlled trial	Public home care patients ≥ 65 years old in five areas in Finland: Forssa, Haapajärvi, Lahti, Juva and Savonlinna	Implicit.<br>Medication review by GP, pharmacist and nurse using SFINX, RENBASE, PHARAO, and Meds75 databases	Mortality, PIMs	6 months
6. Avorn et al 1992	Cluster randomized controlled trial at facility level	Nursing home patients in Massachusetts, USA	Explicit.<br>Education program for physicians whose prescriptions for psychoactive drugs (Benzodiazepines, Hypnotics,	PIMs	2 months

© 2025 Persaud N et al. *JAMA Network Open*.

			Antipsychotics, Antidepressants) exceeded the predefined level		
7. Balsom et al 2020	Randomized controlled trial	Long term care residents > 65 years old in Newfoundland and Labrador, Canada	Implicit.<br>Medication review by pharmacist and pharmacy student focused on identifying medications that were no longer required or potentially harmful	Mortality, Number of medications	6 months
8. Basger et al 2015	Randomized controlled trial	Patients > 65 years old following private hospital discharge in Sydney, Australia	Implicit.<br>Medication review and counselling by clinical pharmacist consisting of medication reconciliation, identification of potential causes of DRPs and recommendations	Mortality, Quality of life	3 months
9. Bayliss et al 2023	Cluster randomized controlled trial at practice level	Outpatient primary care patients ≥ 65 years old in Colorado USA	Implicit.<br>Education program on deprescribing and medication management for both patient and family as well as clinicians. Long-term medications were defined as any medication prescribed for more than 28 days and PIMs were based on the Beers list of drugs to avoid for individuals with cognitive impairment + opioids	Mortality, PIMs	12 months
10. Beer et al 2011	Randomized controlled trial	Community dwelling and LTC residents > 60 years old in Perth Australia	Explicit.<br>Medication review by care provider (GP or treatment specialist) and systematic deprescribing of antihypertensive agents, anti-anginals, diuretics, NSAIDs, and COX-Z inhibitors	Quality of life	4 months
11. Bogaerts et al 2024	Randomized controlled trial	Nursing home residents with moderate to severe dementia and SBP ≤160mmHg in the Netherlands.	Explicit.<br>Treating physicians initiated a stepwise discontinuation of antihypertensive medications over a 6 week period with weekly blood pressure monitoring.	Mortality, PIMs	32 weeks
12. Bosch-Lenders et al 2020	Stepped wedge cluster randomized controlled trial at practice level	Outpatient primary care patients ≥ 60 years old in South Limburg Netherlands	Implicit.<br>Medication review by GP and pharmacist where medications are categorized and checked for indications, side-effects, drug-drug interactions, contraindications, and appropriate dosing. Recommendations are verified with patient's relevant medical specialist prior to implantation.	Quality of life, Number of medications	12 months

© 2025 Persaud N et al. *JAMA Network Open*.

13. Boyd et al 2023	Cluster randomized controlled trial at practice level	Outpatient primary care patients ≥ 65 years old in Colorado, United States	Implicit.<br>The patient component involved mailing educational brochures before primary care visits. The clinician component included monthly deprescribing tip sheets and EHR notifications about upcoming visits with brochure-receiving patients.	Mortality, Hospitalizations, ED visits	4 months
14. Bregnhøj et al 2009	Cluster randomized controlled trial at provider level	Outpatient primary care patients ≥ 65 years old in Copenhagen, Denmark	Implicit.<br>Combined intervention of an interactive educational meeting on polypharmacy for GPs plus feedback on participating patients' medication or single intervention with interactive educational meeting for GPs using MAI index to assess appropriateness	Mortality, Number of medications, PIMs	3 months
15. Briggs et al 2015	Randomized controlled trial	Outpatient emergency department patients > 70 years old in New South Wales, Australia	Explicit.<br>Emergency Department Medication Review by clinical pharmacist using Anticholinergic Burden Scale and Beers Criteria to assess appropriateness	Hospitalizations, ED visits	8 months
16. Brünn et al 2024	Cluster randomized controlled trial at practice level	Adult outpatient primary care patients in Westphalia-Lippe, Germany	Explicit.<br>Physicians conducted annual medication reviews using a CDSS that alerted them to drug interactions, contraindications, and dosing errors. The intervention included physician training and offered remuneration for each review	Mortality, Hospitalizations, PIMs	3.5 years
17. Bryant et al 2010	Cluster randomized controlled trial at provider level	Outpatient primary care patients ≥ 65 years old in New Zealand	Implicit.<br>Medication review by community pharmacists using MAI index to assess appropriateness	Mortality, PIMs	12 months
18. Callegari et al 2022	Cluster randomized controlled trial at facility level	Nursing home residents in Ostfold county, Norway	Explicit.<br>An educational program trained nursing home physicians on using the NorGeP–NH criteria for medication reviews. Physicians attended a 3-hour lecture on pharmacology in older adults, PTD use, and medication reviews with NorGeP–NH. Within two	Quality of life, Number of medications	3 months

© 2025 Persaud N et al. *JAMA Network Open*.

			weeks, they conducted drug chart reviews for enrolled residents.		
19. Campbell et al 2021	Cluster randomized controlled trial at practice level	Outpatient primary care patients ≥ 65 years old in Indiana, United States	Explicit. Computerized decision support tool that alerted providers of high-risk anticholinergics	PIMs	12 months
20. Campins et al 2017	Randomized controlled trial	Outpatient primary care patients ≥70 years old in Barcelona, Spain	Explicit. Medication review by pharmacist using Garfinkel Good Palliative-Geriatric Practice algorithm + STOPP/START criteria	Mortality, Hospitalizaitons, ED visits, Quality of life, medical visits, Number of medications, PIMs	12 months
21. Cateau et al 2021 (IDel)	Randomized control trial	Nursing home residents ≥ 65 years old in Switzerland	Implicit. Medication review by pharmacists using STOPP/START criteria	Mortality, Hospitalizations, Falls	4 months
22. Cateau et al 2021 (QC – DeMo)	Cluster randomized controlled trial at facility level	Nursing home residents ≥ 65 years old in Switzerland	Explicit. Quality circle session focused on deprescribing specific PIMs	PIMs	24 months
23. Clyne et al 2015	Cluster randomized controlled trial at practice level	Outpatient primary care patients ≥70 years old in in Dublin, Ireland	Explicit. Academic detailing by pharmacists and medication review by GP. PIMs identified by a computerized algorithm	Mortality, Quality of life, PIMs	6 months
24. Clyne et al 2016	Cluster randomized controlled trial at practice level	Outpatient primary care patients ≥70 years old in in Dublin, Ireland	Explicit. Academic detailing by pharmacists and medication review by GP. PIMs identified by a computerized algorithm	Mortality, PIMs	12 months
25. Coronado-Vazquez et al 2019	Cluster randomized controlled trial at provider level	Patients ≥ 65 years old, from health centres in Aragon and Andalusia, Spain.	Explicit. Decision support tool by physicians and nurses providing information about associated risk of inappropriate medications using START/STOPP criteria.	PIMs	6 months
26. Crotty et al 2004	Cluster randomized controlled trial at provider level	Patients in 10 nursing homes in southern Adelaide, Australia	Implicit. Medication review: two multidisciplinary case conferences involving the resident’s general practitioner, a geriatrician, a pharmacist and residential care staff.	Mortality, Number of medications, PIMs	3 months

© 2025 Persaud N et al. *JAMA Network Open*.

27. Del Cura-González et al 2022	Cluster randomized controlled trial at practice level	Patients aged 65 – 74 with at least 3 chronic conditions from primary care practices in Spain.	Implicit.<br>Family physicians completed a 4-week online course on multimorbidity, polypharmacy, prescribing, adherence, and shared decision-making, followed by structured patient medication reviews.	ED visits, Non-serious adverse drug events, Quality of life, Medical visits, PIMs	12 months
28. Desborough et al 2020	Cluster randomized controlled trial at facility level	Patients ≥ 65 years old, from care homes in the East England	Implicit.<br>Multi-professional (general practitioner, clinical pharmacist) medication review.	Mortality, Hospitalizations, Falls	12 months
29. Dugre et al, 2021	Cluster randomized controlled trial at facility level	Patients at 8 residential aged care facilities in Australia.	Implicit.<br>One-off application of the Medication Regimen Simplification Guide for Residential Aged CarE: structured tool to assist pharmacists and other clinicians to identify opportunities for medication simplification.	Mortality	12 months
30. Elliott et al 2017	Randomized controlled trial	Patients ≥ 50 years old, from one hospital based home health agency in Searcy, Arkansas, US.	Implicit.<br>Medication review. Pharmacist reviewed drug-drug, drug-gene, and cumulative drug and/or gene interactions using the YouScript CDST to provide drug therapy recommendations to clinicians	Hospitalizations, ED visits	12 months
31. Erler et al 2012	Cluster randomized controlled trial at practice level	Patients ≥ 70 years old with chronic kidney disease, from primary care practices in Germany	Explicit.<br>Individual dose adjustment<br>1) interactive 1-hour workshop for physicians on detection and management of CKD, 2) provision of a desktop checklist of medications to be reduced or avoided in patients with CKD, 3) provision of patient information leaflets, and 4), training in the use of the software DOSING	PIMs	6 months
32. Etherton-Beer et al 2023	Randomized controlled trial	Patients ≥ 65 years old living at residential aged care facilities in Sydney and Perth Australia	Explicit.<br>The intervention involved research pharmacists creating a deprescribing plan using an algorithm, prioritizing medications with the least benefit and withdrawal risk. GPs approved the plan before implementation.	Mortality, Hospitalizations, Quality of life, Falls, Number of medications	12 months

© 2025 Persaud N et al. *JAMA Network Open*.

33. Fournier et al 2020	Cluster randomized controlled trial at facility level (post-hoc analysis)	Frail nursing home residents in Belgium.	Implicit.<br>Medication review: application of STOPPFrail criteria.	PIMs	8 months
34. Frankenthal et al 2014	Randomized controlled trial	Patients ≥ 65 years old, at a chronic care geriatric facility in central Israel.	Explicit.<br>Screening medications with STOPP/ START criteria followed up with recommendations to the chief physician.	Mortality, Hospitalizations, Quality of life, Falls, Number of medications, PIMs	12 months
35. Fried et al 2017	Randomized controlled trial	Veterans ≥ 65 years old, from primary care clinics at a VA<br>Connecticut Medical Center, in US.	Explicit.<br>Computerized Software Tool to Reduce Inappropriate Medications (TRIM): extracts medications and chronic conditions from the EHR and contains data entry screens for information obtained from brief chart review and telephonic patient assessment. Using Beers and STOPP criteria	Number of medications, PIMs	13 months
36. Furniss et al 2000	Cluster randomized controlled trial at facility level	Nursing home residents in Manchester, UK	Implicit.<br>Medication review conducted by study pharmacist.	Mortality, Number of medications	8 months
37. Garcia-Gollarte et al 2014	Cluster randomized controlled trial at provider level	Physicians and patients ≥ 65 years old living at least 3 months in the nursing home, from 37 nursing homes owned by a private company in Spain.	Implicit.<br>Education program: 10 hours education program for physicians followed by on-demand prescription support by phone. Using STOPP-START criteria	Hospitalizations, ED visits, Falls, Medical visits, Number of medications, PIMs	12 months
38. Gedde et al 2020	Cluster randomized controlled trial at facility level.	Patients aged ≥65 years with at least 2 weeks of residency in thirty-three Norwegian nursing<br>homes	Implicit.<br>COSMOS Intervention: Communication and advanced care planning, Systematic pain management, Medication reviews with collegial mentoring, Organization of activities adjusted to the individuals' need and preferences, and Safety.	Number of medications, PIMs	4 months
39. Gedde et al 2022	Stepped wedge cluster randomized controlled trial	Home-dwelling patients aged ≥65 years with dementia and regular psychotropic drug use in Norway.	Implicit.<br><br>LIVE@Home.Path included education on dementia, assistive technology	Number of medications	6 months

© 2025 Persaud N et al. *JAMA Network Open*.

	at individual level		assessments, volunteer support via organizations like the Red Cross, and empowering patients by connecting with GPs for care planning and medication reviews.		
40. Green et al 2024	Pilot randomized controlled trial	Primary care patients aged ≥65 years living with dementia in Baltimore and Denver, United states	Implicit.<br>The ALIGN intervention is a pharmacist-led telehealth deprescribing program, including an educational brochure, a video/phone consultation to review medications, and tailored recommendations sent to the primary care provider via the EHR.	Number of medications	3 months
41. Guerts et al 2015	Randomized controlled trial	Patients ≥ 60 years old, use ≥5 drugs with at least one for a cardiovascular disorder. In 8 primary care settings in Netherlands.	Implicit.<br>Clinical Medication Review (CMR) + Web based pharmaceutical care plan (PCP) developed by the pharmacist and GP. It included 3 components: identifying DRPs and PCIs, proposed care interventions, and implementation.		18 months
42. Haag et al 2016	Randomized controlled trial	Patients ≥60 years old, in transition from hospital to independent living. In a primary care work group at tertiary care academic medical center in midwestern US.	Implicit.<br>Medication Review using STOPP/START criteria; MAI Index; MMAS:<br>1) Pharmacists conducted telephone consultations with patients after hospital discharge<br>2) Pharmacists used EMRs to review all medications and identify DRPs<br>3) Pharmacists made evidence-practice based recommendations<br>4) Recommendations communicated to the patient's care team provider via EMR	Mortality, Hospitalizations, ED visits, Number of medications, PIMs	11 months
43. Haider et al 2023	Cluster randomized controlled trial at facility level	Residents from residential aged care facilities in ACT, Australia	Explicit.<br>Pharmacists joined the interdisciplinary care team, conducting medication reviews, reconciliation during transitions, clinical audits, and providing education to staff, residents, and families.	Number of medications, PIMs	12 months
44. Hanlon et al 1996	Randomized controlled trial	Patients ≥65 years old, with evidence of polypharmacy and received primary care in	Implicit.<br>Medication review: a pharmacist reviewed each patient's medical record and	Mortality, Non-serious adverse drug events, Quality	12 months

© 2025 Persaud N et al. *JAMA Network Open*.

		the general medical clinic of Veterans Affairs, in the United States	medication list. Then formulated recommendations to the patient's primary physician. Education program: the pharmacist educated the patient regarding drug-related problems and changes.	of life, Number of medications, PIMs	
45. Harnisch et al 2024	Randomized controlled trial	Patients with dementia both living in nursing homes and community dwelling and their primary care physicians in the United States	Explicit.<br>The intervention involved sending a series of three overprescribing warning letters to high-volume primary care physician prescribers of the antipsychotic quetiapine. These letters informed the PCPs that their prescribing rates were high compared to their peers and were under review by Medicare	Mortality, PIMs	3 months
46. Herrinton et al 2023	Randomized controlled trial	Patients ≥76 years old, who used 10 or more prescription medications, from pharmacies in the United States	Implicit.<br>Medication review: Pharmacists offered a medication review, in which they assessed current and past medications, discussed medication adherence, patients' experiences with the drug, and patient preferences. The patient and pharmacist also made a shared decision to prioritize deprescribing.	Hospitalizations, Number of medications	6 months
47. Holdhus et al 2019	Randomized controlled trial	Patients ≥18 years old, from three medical wards at Akershus University Hospital, Norway.	Implicit.<br>Medication reconciliation: discrepancies in the medication list at primary care services<br>within 10 days after discharge compared with<br>the medication list at discharge from hospital.	PIMs	20 months
48. Holland et al 2023	Cluster randomized controlled trial at the pharmacist level	Home care residents ≥65 years old, taking at least one prescribed drug, in England, Scotland, and Northern Ireland	Implicit.<br>Medication review and reconciliation: Each prescriber provided pharmaceutical care, with weekly visits. Prescribers developed a care plan for each resident, did medicine review/reconciliation, trained staff, and supported with medicines related procedures, deprescribing, and authorization of prescriptions.	Falls, Quality of life, Hospitalizations, Mortality	6 months

© 2025 Persaud N et al. *JAMA Network Open*.

49. Johansson et al 2023	Randomized controlled trial	Patients with type 2 diabetes, taking at least twelve medications, in Denmark.	Implicit.<br>Medication review: Patients underwent a medication consultation conducted by a physician. The consultation involved a medication review that aimed to facilitate appropriate pharmacotherapy by a systematic and critical review of the medications where the indication, risk–benefit ratio and potential drug–drug interactions for each treatment were evaluated.	Hospitalizations, Number of medications, Quality of life, PIMs	6 months
50. Jungo et al 2023	Cluster randomized controlled trial at the provider level	Patients ≥65 years old, with three or more chronic conditions and five or more long-term medications, from primary care, in Switzerland.	Implicit.<br>Computerized decision support tool: The intervention to optimise pharmacotherapy centred around an electronic clinical decision support system was conducted by general practitioners, followed by shared decision making between general practitioners and patients, and was compared with a discussion about medication in line with usual care between patients and general practitioners	Falls, PIMs	
51. Junius-Walker et al 2021	Cluster trial at patient level	Patients ≥65 years old, from nursing homes in Germany	Implicit.<br>1) Medication Review with pharmacist<br>2) Educational sessions for GPs and nurses<br>3) drug safety toolbox for HCPs (materials to promote collaboration & look up drug-related risks)<br>4) change management seminars for GPs, PhmD, nurses	Mortality, Hospitalizations, ED visits, Quality of life, Falls, PIMs	6 months
52. Juola et al 2015	Cluster randomized controlled trial at ward level.	Patients ≥65 years old, in assisting living facilities, using at least 1 medication, with estimated life expectancy of over 6 months. In Helsinki, Finland.	Explicit.<br>Educational intervention for nursing staff: two 4h interactive training sessions based on constructive learning theory to recognize harmful medications and adverse drug events.	Falls, PIMs	12 months
53. Kirkham et al 2020	Stepped wedge cluster randomized	Interdisciplinary staff at 10 Canadian LTC facilities.	Implicit.<br>Educational in-service 90-min session delivered in person by study investigators	Falls, PIMs	15 months (12 months of follow up and 3

© 2025 Persaud N et al. *JAMA Network Open*.

	controlled trial at facility level		at each LTC facility, provision of evidence-based tools to assess and monitor NPS, and monthly interdisciplinary LTC team meetings to discuss individuals for whom antipsychotics could be reduced or discontinued.		months of pre-implementation period)
54. Köberlein-Neu et al 2016	Stepped wedge cluster randomized controlled trial at provider level	Primary care outpatients over 65 yrs old with three chronic diseases and five or more long term medications.	Implicit.<br>Medication management and care provided by homecare specialists. Pharmacist undertook a comprehensive medication review and sent letter of recommendation to home-care specialist, who then added info to patients' homecare situation, then sent this to PCP.	Quality of life, PIMs	15 months
55. Könner et al 2015	Cluster randomized controlled trial at facility level.	Nursing home residents over 65yrs old, spent ≥3months in facility, had no/mild cognitive impairment. In 12 nursing homes in Berlin, Germany.	Implicit.<br>Online 5-6h course, offered for 6 weeks to the GPs of the intervention group, while the GPs of the control group could participate after data collection. Participants received Continuing Medical Education (CME) points after completing the course. Nursing home staff also provided with 1-day seminars.	PIMs	10 months
56. Kornholt et al 2022	Randomized controlled trial	Geriatric outpatients taking ≥9 medicines, at Copenhagen University Hospital, Frederiksberg, Denmark.	Implicit.<br>An additional medication consultation with a physician from the Department of Clinical Pharmacology focusing on reviewing the medication, i.e., ensuring the appropriateness of all prescriptions, informing the patient about their medicines, and ensuring enhanced cross-sectoral communication and collaboration.	Mortality, Hospitalizations, Quality of life, Falls, Number of medications	13 months
57. Kornholt et al 2023	Randomized controlled trial	Geriatric outpatients taking ≥9 medicines, at Copenhagen University Hospital, Frederiksberg, Denmark.	Implicit.<br>The intervention was a physician-led medication review in which clinical pharmacologists reviewed patients' medical and medication histories, identified potentially inappropriate prescriptions using tools like the Drug Burden Index. Proposed changes were discussed with the patient's general practitioner and geriatrician, then	Number of medications	13 months

© 2025 Persaud N et al. *JAMA Network Open*.

			implemented during an in-person consultation with patient consent. Follow-up occurred at 4 and 13 months as part of usual care.		
58. Kouladjian O'Donnell et al 2021	Cluster randomized controlled trial at provider level.	Patients ≥65 years old, who were referred to a participating pharmacist for an HMR (post-randomization) were screened and invited by the pharmacist to participate. In Australia	Implicit.<br>Pharmacists producing G-MEDSS (Goal-directed Medication Review Electronic Decision Support System) reports about their HMR (Home Medicine Reviews) patients to send with the HMR report to the patient's GP and providing the patient/carer G-MEDSS reports.	Mortality, Falls, Medical visits, Number of medications	3 months
59. Krska et al 2001	Randomized controlled trial	Patients >65 years old, with at least 2 chronic disease states and taking at least 4 prescribed drugs regularly. In the Grampian region of Scotland.	Implicit.<br>Pharmacist completed a patient profile. A medication plan was drafted for each pt of the intervention group, with all potential/actual "pharmaceutical care issues (PCI)", with actions planned. Copies in the pt's medical notes and given to GP. GP asked to indicate level of agreement with each PCI. Pharmacist implemented agreed actions.	ED visits, Medical visits	3 months
60. Kua et al 2021	Stepped-wedge cluster randomize controlled trial at ward level.	4 Nursing home residents >=65 years old, taking >=5 medications. In Singapore.	Explicit.<br>Pharmacist-led 5-step team-care deprescribing intervention:<br>1. Med review by pharmacist<br>2. check for interactions (drug-drug or drug-food)<br>3.discuss deprescribing with nurses for each resident<br>4. nurses communicated with physicians for reviewing and deprescribing decisions<br>5. agreed action plans documented and follow-ups	Mortality, Hospitalizations, Falls, Number of medications	12 months
61. Kwint et al 2011	Randomized control trial	Patients >=65 years old, use ≥5 drugs with at least one being dispensed from automated system, from six Dutch community pharmacies. In Netherlands	Implicit.<br>Patient data from community pharmacy and GP collected by pharmacist and included drug dispensing records, reviewed using Dutch treatment and prescription guidelines	Mortality, PIMs	6 months

© 2025 Persaud N et al. *JAMA Network Open*.

			and implicit criteria. Pharmacists sent report to GP to be discussed.		
62. Lampela et al 2010	Randomized controlled trial	Home dwelling subpopulation of the larger GeMS (Geriatric Multidisciplinary Strategy for Good Care of the Elderly) study, >= 75 years old (random). In eastern Finland.	Explicit.<br>Comprehensive geriatric assessment by 2 MDs, 2 RNs, 2 physiotherapists and 1 nutritionist: Adjustment of medication, evaluation of indications for all drugs in use, withdrawn of drugs without an indication, clinical examination.	Mortality, Number of medications, PIMs	12 months
63. Lenaghan et al 2007	Randomized controlled trial	Patients >=80 years living at home and taking ≥4 oral daily meds. And one of the following: living alone; record of confused mental state, vision or hearing impairment; prescribed medicines associated with medication-related morbidity; or prescribed >7 regular oral medicines. In rural north Norfolk, England.	Implicit.<br>Two home visits by a community pharmacist who educated the patient/carer about their medicines, noted any pharmaceutical care issues, assessed need for an adherence aid, and subsequently met with the lead GP to agree on actions.	Mortality, ED visits, Quality of life, Number of medications	6 months
64. Lenander et al 2014	Randomized controlled trial	Patients >=65 years with 5+ medications, from Liljeholmen Primary Care Centre, Stockholm, Sweden.	Explicit.<br>Patients answered a questionnaire regarding medications. The pharmacist reviewed all medications (prescription, non-prescription, and herbal) regarding recommendations and renal impairment, giving advice to patients and GPs. Each patient met the pharmacist before seeing their GP.	Hospitalizations, Non-serious adverse drug events, Medical visits, Number of medications	15 months
65. Lexow et al 2022	Randomized controlled trial	Long term care facility residents ≥ 65 years old in Leipzig, Germany	Implicit.<br>Pharmacists performed medication reviews and gave recommendations to the GP	Mortality, Hospitalizations, Falls, PIMs	6 months
66. Lin et al 2018	Randomized controlled trial	Patients >=65 years old, with >=3 chronic diseases and, >=6 prescription items, had >4 outpatient visits/visited 2x+ the study hospital in the	Implicit.<br>Comprehensive intervention elements provided by clinical pharmacist for patients in the MTM (medication therapy management) group, through direct patient contact, physician contact, or	Mortality, Quality of life	16 months

© 2025 Persaud N et al. *JAMA Network Open*.

		preceding year. At the outpatient clinics of China Medical University Hospital Taiwan	comprehensive medical chart review. Once action plan decided, pharmacist contacted the patient's physicians.		
67. Liou et al 2021	Randomized controlled trial	Patients >= 75 years old, prescribed 8+ medications., from a Veterans Administration nursing home in Taiwan.	Implicit.<br>Pharmacists completing medication administration record, assessing medication appropriateness of prescriptions, surveying utilization of healthcare resources, and identifying DRPs in the intervention group.<br>Afterwards: educate patients on medication administration and provide tools to improve administration/compliance (7-day dosing aid, pill splitter, health journal).	Mortality, Hospitalizations, ED visits, Quality of life, Medical visits, Number of medications, PIMs	24 months
68. Liu et al 2023	Randomized controlled trial	Patients ≥ 45 years old living in California, Nebraska, or Iowa with Medicare or Medicaid insurance and have a primary caregiver.	Implicit.<br>Telephone-delivered medication review by an interprofessional care team	Number of medications, PIMs	12 months
69. Mahlknecht et al 2021	Cluster randomized controlled trial at provider level	Primary care setting. Involved GPs and >=75 years old community-living patients in the province of Bolzano, Italy.	Explicit.<br>Review of patient's medication regimens by three experts who gave specific recommendations for drug discontinuation.	Mortality, Hospitalizations, Quality of life, Falls, Number of medications	24 months
70. Malet-Larrea et al 2016	Cluster randomized controlled trial at practice level	In 178 community pharmacies in four Spanish provinces (Guipuzcoa, Granada, Las Palmas and Tenerife)	Implicit.<br>Medication review. Pharmacists in the intervention group received a 3-day training course covering clinical management of aged patients, the MRF method, communication with patients and doctors, study protocol and documentation forms.<br>Medication Review with follow-up provided in community pharmacies: to detect drug-related problems to identify, prevent and solve negative clinical outcomes.	Hospitalizations	6 months
71. Martin et al 2018	Cluster randomized	Patients aged 65 years	Explicit.	Mortality, PIMs	6 months

© 2025 Persaud N et al. *JAMA Network Open*.

	controlled trial at practice level	and older who were prescribed 1 of 4 Beers Criteria medications (sedative-hypnotics, first-generation antihistamines, glyburide, or nonsteroidal anti-inflammatory drugs), recruited by Community pharmacists in Quebec, Canada.	Pharmacists in the intervention group were encouraged to send patients an educational deprescribing brochure in parallel to sending their physicians an evidence-based pharmaceutical opinion to recommend deprescribing.		
72. McCarthy et al 2022	Cluster randomized controlled trial at practice level	Patients ≥65 years old and prescribed ≥15 repeat medicines, from 51 general practice clinics in Ireland	Implicit.<br>SPPiRE medication review: (Supporting Prescribing in Older Adults with Multimorbidity in Irish Primary Care) GPs received login info to a website with 5 training videos and instructions on the SPPiRE medication. Videos: background information on multimorbidity and polypharmacy, PIP, eliciting pt treatment priorities, and a brown bag medication review. GPs instructed to book a double appointment and ask patients to bring medicines to the medication review visit.	Mortality, Hospitalizations, ED visits, Quality of life, Medical visits, Number of medications, PIMs	12 months
73. Milos et al 2013	Randomized controlled trial	Patients >=75 years old, from community/primary nursing homes in Sweden.	Explicit.<br>Medication reviews performed by trained clinical pharmacists based on nurse-initiated symptom assessments with team-based or distance feedback to the physician.	Mortality, Number of medications, PIMs	2 months
74. Milos et al 2021	Randomized controlled trial	Patients ≥75 years old, from community/primary nursing homes in Sweden	Explicit.<br>Pharmacists performed a systematic medication review based on symptom assessment made by a nurse with good knowledge of the patient.	Mortality, Hospitalizations	12 months
75. Moga et al 2017	Randomized controlled trial	Patients >=65 years old taking >=1 anticholinergic drug, at University of Kentucky's Alzheimer's Disease Center, US.	Implicit.<br>Pharmacist provided a revised medication plan based on the drug review, which was discussed with the participant and/or their Legally Authorized Representative. Plan: to	Quality of life, PIMs	2 months

© 2025 Persaud N et al. *JAMA Network Open*.

			recommend discontinuation or replacement of any potentially inappropriate drug with anticholinergic properties, with safer drug alternatives. Pharmacist educated pts on medication safety, and MD made recommended medication changes.		
76. Mortsiefer et al 2021	Cluster randomized controlled trial at practice level	Patients ≥70 years old with frailty syndrome from primary care clinics in Germany	Implicit.<br>GPs received education sessions on deprescribing. GP-led family conferences for shared decision making involving the participants, family caregivers, and nursing services were held in the patients' homes were performed, in which a there was a medication check.	Hospitalizations, Number of medications	
77. Muth et al 2018	Cluster randomized controlled trial at practice level	Patients ≥60 years old, ≥3 chronic conditions under pharmacological treatment, and ≥5 long-term drug prescriptions with systemic effects, from 72 general practices in Hesse, Germany	Implicit.<br>Healthcare assistant conducted a checklist-based interview with pts on medication-related problems and reconciled their medications. Assisted by a computerized decision support system, the GP optimized medication, discussed with patients and adjusted.	Hospitalizations, Quality of life, Number of medications	9 months
78. Navy et al 2018	Randomized controlled trial	Patients >=65 years old, continuous members of KPCO health center for 3 months prior, members of Denver/Boulder metropolitan area residing at home, with current supply of alprazolam, in US.	Explicit.<br>Educational outreach regarding alprazolam use reduction via mailed letter. Patients/caregivers requested to call the pharmacist to discuss reduction of alprazolam use. Alternative treatment options were discussed.	PIMs	6 months
79. Olsson et al 2012	Randomized controlled trial	Patients >=75 years old, taking >=5 drugs, living at home, recently discharged from hospital, in municipality of Örebro, Sweden,	Explicit.<br>Control: home visit by urse within one month after discharge, QoL survey by post at six months, and second home visit by study nurse at 12 months.<br>Intervention A: control + letter with prescription review sent to physician/primary care center.<br>Intervention B: A + current and comprehensive medication record	Mortality, Quality of Life, Number of medications	12 months

© 2025 Persaud N et al. *JAMA Network Open*.

			consisting of the patient's written drug regimen and indications sent to pt to enable participation in his/her drug treatment. Accompanied by instruction to utilize the record and discuss drug treatment with physicians.		
80. Parker et al 2019	Randomized controlled trial	Patients >=65 years old, from from three nephrology centers (Akershus University Hospital; Oslo University Hospital, Ullevål; and Vestre Viken Hospital Trust, Drammen), in Norway.	Explicit.<br>Recommended medication changes added to EMR, with STOPP/START criteria. The attending physician could choose to implement the recommendations or not. For the control group, no notifications of recommendations were recorded, but in cases of severe inappropriateness, the investigator would note such in the medical record or inform the attending physician.	Mortality, PIMs	6 months
81. Patterson et al 2010	Cluster randomized controlled trial at facility level	Patients >=65 years old, from long-term care (nursing) homes, in Northern Ireland.	Explicit.<br>Education program: specially trained pharmacists visited intervention homes monthly for 12 months and reviewed residents' clinical and prescribing information, applied an algorithm that guided them in assessing the appropriateness of psychoactive medication, and worked with GPs to improve the prescribing of these drugs.	Mortality, Falls, PIMs	12 months
82. Phelan et al 2024	Cluster randomized controlled trial at practice level	Patients ≥60 years old from primary care clinics in Washington, United States	Implicit.<br>Patients received educational brochures and handouts via mail. Decision support tool was sent to clinicians to help initiate conversations about deprescribing.	Falls	18 months
83. Pitkälä et al 2014	Cluster randomized controlled trial at ward level.	Patients >=65 years old; living permanently in an assisted living facility; using at least 1 medication; having an estimated life expectancy >6 months. Residents in 20 wards of assisted living facilities in Helsinki, in Finland.	Explicit.<br>Two 4-hour interactive training sessions for nursing staff based on constructive learning theory to recognize potentially harmful medications and corresponding adverse drug events. Nurses who identified medication-related problems were to bring those to the consulting physician's	Mortality, Hospitalizations, Quality of life, Medical visits, PIMs	12 months

© 2025 Persaud N et al. *JAMA Network Open*.

			attention, who decided whether to change or continue a medication.		
84. Pitkälä et al 2001	Randomized controlled trial	All home care patients in a rural area, Kirkkonummi-Siuntio, in Finland.	Implicit.<br>Medications of all participants were reviewed and counted during an in-home assessment by a home nurse. Necessary revisions (dose reduction, discontinuation, possible additions) were performed through the tight monitoring of day hospital care and with the pt. Pts attended day hospital for 2-3 days/week for 2 months. Hospital physician would make the recommendations to medications. Follow-up for 10 months after completion of the 2month intervention	Number of medications	12 months
85. Potter et al 2016	Randomized controlled trial	Patients >=65 years old, living in four residential aged care facilities in rural mid-west Western Australia	Explicit.<br>Individualized medicine review followed by the planned cessation of non-beneficial medicines.	Mortality, Hospitalizations, Quality of life, Falls, Medical visits, Number of medications	12 months
86. Raebel et al 2007	Randomized controlled trial	Patients >=65 years old, from community practice sites (in the ambulatory care environment at KPCO: Kaiser Permanente Colorado), in US	Explicit.<br>Computerized software/System alert: Rx and age information were linked to alert pharmacists when patients were prescribed one of 11 PIMs. Rx label was able to be printed only after pharmacist reviewed Rx and determined appropriateness. When an intervention patient was newly prescribed a potentially inappropriate medication, the pharmacist was notified via the alert generated from PIMS. The medication alert functioned by not allowing prescription label to print until pharmacist had actively intervened to determine whether the prescription should be dispensed.	PIMs	12 months
87. Rankin et al 2022	Cluster randomized controlled trial	Community-dwelling patients ≥ 70 years old from primary care practices in Ireland.	Explicit.<br>Intervention GPs received access to an online video and supplementary materials.	Quality of life, PIMs	9 months

© 2025 Persaud N et al. *JAMA Network Open*.

	at practice level		They then completed medication reviews with recruited patients using STOP/START criteria.		
88. Richter et al 2019	Cluster randomized controlled trial at facility level	Residents of nursing homes, excluding those with primary diagnosis of schizophrenia or bipolar disorder or patients temporary staying in respite care. In Germany.	Explicit.<br>Medication Review + Education program for staff. Physicians specialized in psychotropic drug reviewed the medications of all patients (intervention and control), at baseline and after 3, 6 and 9 months. For nursing homes in the intervention, selected staff were trained (2-day workshop) and instructed to work as experts for person-centered care. Staff nursing homes attended a 60 min info session about the study.	Quality of life, Falls	12 months
89. Rieckert et al 2020	Cluster randomized controlled trial at practice level	Patients aged 75+ taking >/= 8 medications, from general practice clinics in Austria, Germany, Italy, UK	Implicit.<br>Computerized decision support tool: comprehensive medication review (CMR) based on patient data and evidence. The PRIMA-eDS tool consisted of the electronic case report form and the CMR. Patient data entered in the electronic case report form to analyze and generate a CMR, providing advice for drug discontinuation or modification. Decisions at discretion of the GP and pt. GPs were instructed to use the electronic decision support tool directly after randomization, and at all 3 follow-up consultations.	Mortality, Hospitalizations, Quality of life, Falls, Number of medications	24 months
90. Rognstad et al 2013	Cluster randomized controlled trial at practice level	Norweigan GPs and patients aged ≥70, from general practice clinics.	Explicit. Education Program: educational package on safer prescribing. 2-day pre-study training sessions, focusing on: (1) safety issues in relation to pharmacological treatment in older people.<br>(2) the rationale for the 13 listed PIPs. (3) how to facilitate learning within a group setting.	PIMs	6 months
91. Romskaug et al 2020	Cluster randomized controlled trial	Norweigan GPs and patients >=70 years old, taking >= 7	Implicit.<br>Medication Review + Education Program, Multi-step: (1) geriatric assessment of pts +	Hospitalizations, Quality of life	6 months

© 2025 Persaud N et al. *JAMA Network Open*.

	at provider level	medications administered by home nursing services.	medication review. (2) meeting between geriatrician and GP to discuss medication and care plan. (3) GP follow-up		
92. Roughead et al 2022	Randomized controlled trial	Residents of aged-care facilities taking four or more medications in Australia.	Implicit.<br>Pharmacists reviewed medicine charts to identify any concerns.	Quality of life	12 months
93. Rudolf et al 2021	Cluster randomized controlled trial at practice level	Physicians from the practice networks of Witten/Herdecke University and Hannover Medical<br>School, and patients >=70 years old, taking > 5 drug agents for regular, long-term use, in Germany.	Explicit.<br>Medication Review + Education program: GPs in the intervention receive training workshops and materials, including a PRISCUS card and manual, with a hotline for consultation with a clinical pharmacologist. Practices randomized to team training involve medically trained team members receiving adapted materials.	Mortality, Hospitalizations, Quality of life, PIMs	12 months
94. Sacarny et al 2018	Cluster randomized controlled trial at provider level	Primary care practitioners (PCPs)<br>or prescribers chosen by a CMS (Centers for Medicare & Medicaid Services) analysis of quetiapine prescribing inMedicare PartD (prescription drug coverage). In US.	Explicit.<br>Audit & Feedback: mailed peer comparison letters that indicated that the prescriber's quetiapine prescribing was under review by CMS and was extremely high relative to the within-state peers. The letter discussed that high quetiapine prescribing could be appropriate but was concerning for medically unjustified use, and encouraged GPs to review their prescribing patterns and explained that they could expect to receive.	PIMs	9-24 months
95. Schäfer et al 2017	Cluster randomized controlled trial at practice level	GPs recruited patients ages 65-84 with >=3 chronic conditions. In Hamburg, Düsseldorf and Rostock, Germany.	Implicit.<br>Meetings with patients including medication review. Within the intervention, GPs had three 30 min talks with each of their pts in addition to routine consultations. First talk: aimed at identifying treatment targets and priorities of the pt. Second talk: medication taken by the pt was discussed based on a 'brown bag' review of all the medications the pt had at home (pts take all their medication in the office). Third talk: to	Hospitalizations, Quality of life, Medical visits, Number of medications	12 months

© 2025 Persaud N et al. *JAMA Network Open*.

			discuss goal attainment and future treatment targets.		
96. Schmidt-Mende et al 2016	Cluster randomized controlled trial at provider level	Patients >=65 years old, from general practice clinics located in Stockholm's County with list size greater than or equal to 3000. In Sweden	Implicit.<br>Educational sessions developed by 2 GPs, 3 pharmacists, and a study nurse consisting of: 1. a PowerPoint with theoretical knowledge on PIMs based on the national indicators and feedback on prescribing of PIMs, 2. local consensus process with interprofessional discussion and a proposed procedure for the performance of medication reviews, and 3. a reminding education session after 4 months.	Mortality, Hospitalizations, ED visits, Medical visits, PIMs	9 months
97. Schmidt et al 1998	Cluster randomized controlled trial at facility level	Long-term care residents from nursing homes in Sweden.	Explicit.<br>Medication review: regular (~1/month) multidisciplinary team meetings between pharmacists, physicians and nursing personnel to discuss participants drug use.	PIMs	12 months
98. Sellors et al 2003	Cluster randomized controlled trial at provider level	Patients >=65 years old, taking >=5 medications, in family practices in 24 sites in Ontario, Canada.	Implicit.<br>Medication review: pharmacist-led face-to-face medication reviews with patients and then sent written recommendations to physicians based on any drug related problems. Pharmacist and physician met at 3 and 5 months to discuss implementation. Pharmacist monitored patients drug therapy by conducting semi structured interviews via phone at 1 and 3 months.	Number of medications	5 months
99. Sheppard et al 2024	Randomized controlled trial	Patients aged ≥ 80 prescribed more than 2 HTN drugs from primary care practices in England.	Explicit.<br>Participants in the intervention group had 1 antihypertensive drug withdrawn from their medication regimen.	Mortality, Hospitalizations, Number of medications	
100. Strauven et al. 2019	Cluster randomized controlled trial at facility level.	Patients aged ≥65, not receiving palliative care, under care of participating GP, in nursing homes in Belgium.	Implicit.<br>Education + Medication review, including 3 components: a blended training program, local interdisciplinary meetings, and interdisciplinary case conferences (ICC). ICCs involving the GP, pharmacist, and	PIMs	15 months

© 2025 Persaud N et al. *JAMA Network Open*.

			nurse were the key component (structured and repeated interdisciplinary face-to-face medication reviews every 4 months, ie, 3 times over a 12-month period).		
101. Sultan et al 2022	Randomized controlled trial	Patients registered at a geriatric outpatient clinic in The Netherlands.	Explicit.<br>A blinded review panel independently scored the number of potentially inappropriate medicines using STOPP/START criteria.	PIMs	6 weeks
102. Syafhan et al 2021	Randomized controlled trial	Patients recruited sequentially, according to risk stratification, i.e. Stratum 1: >=18 years old with at least one unplanned hospital admission or two or more A&E attendances in the previous 12 months, and prescribed at least 6 regular oral or inhaled, long-term medicines. Stratum 2: patients >=18 years old, prescribed at least 10 regular oral or inhaled, long-term medicines. In 4 UK regions, with 2 GP practices in each region.	Implicit.<br>Medication Review: pharmacists conducted reviews of patient's medical history, records, and lab data, and met patients to compile medication histories, assess adherence, and the appropriateness of medications. Pharmacists created lists of potential MRPs and developed individual pt plans. Where appropriate the pharmacist facilitated referrals to other healthcare professionals and provided GPs with a report of actions taken and further recommendations. Visits at 2 and 4 months.	Hospitalizations, Medical visits, PIMs	6 months
103. Tadrous et al 2020	Cluster randomized controlled trial at facility level	Nursing home residents, in Ontario, Canada.	Implicit.<br>Education program: academic detailing delivered by health professionals who arranged meetings (with administrators, physicians, pharmacists, nurses, and support workers), presentations, group visits (with 2-6 clinicians), and 1-on-1 visits (traditional academic detailing visits).	Hospitalizations, ED visits, Falls	12 months
104. Tamblyn et al 2003	Cluster randomized controlled trial at provider level	GP >=30 years old, who had practices in Montreal, spent at least 70% of the week in private fee-for-service practice and had a minimum of 100 elderly patients.	Explicit.<br>Computerized decision support: computerized software alert identifying any of 159-drug related problem and suggesting alternative therapy.	PIMs	13 months

© 2025 Persaud N et al. *JAMA Network Open*.

		Patients >=66 years old, who had been seen on 2 or more occasions by the study physician in the past year and living in the community. In Quebec, Canada			
105. Tannenbaum et al 2014	Cluster randomized controlled trial at practice level.	Long-term users of benzodiazepine medication aged 65-95 years with a minimum of 5 active prescriptions. 30 community pharmacies in the greater Montreal area, in Canada.	Explicit.<br>Deprescribing patient empowerment intervention: booklet informing pts about the risks of benzodiazepines, knowledge statements designed to create cognitive dissonance about the safety of benzodiazepine use, education about drug interactions, suggestions for equally of more effective alternatives, tapering recommendations.<br>It asks participants to discuss the deprescribing recommendations with their physician and/or pharmacist.	PIMs	6 months
106. Terrell et al 2009	Cluster randomized controlled trial at provider level.	Emergency physicians (emergency medicine faculty and<br>resident physicians), and patients >=65 years old being discharged from the ED, in<br>Indianapolis, Indiana, US.	Explicit.<br>Computerized decision support: Decision support that advised against use of 9 potentially inappropriate medications and recommended safer substitute therapies	PIMs	30 months
107. Toivo et al 2019	Cluster randomized controlled trial at facility level.	Patients >=65 years old receiving regular home care, using at least one prescription medicine. in Primary Care in Lohja, Finland: all 5 home care units, the public healthcare center, and a private community pharmacy.	Explicit.<br>Coordinated medication risk management (CoMM):<br>1. Triage Meetings: Physicians, pharmacists, and nurses discuss actions for home care pts, review medications and identify potential DRPs.<br>2. Prescription Review: pharmacists identify potential DRPs using computerized tools, and report those clinically significant to physicians.<br>3. Comprehensive Medication Review: Pharmacists use medication lists, medical	Number of medications, PIMs	12 months

© 2025 Persaud N et al. *JAMA Network Open*.

			records, and DRP risk assessments to make recommendations. Nurses deliver written medication review reports to physicians, who discusses recommendations with the pharmacist.<br>4. Clinical Medication Review (CMR): trained pharmacists conduct CMRs for pts with complex medication regimens and prepare case reports with recommendations for physicians.<br>5. Physician Decision-Making: Physicians make final decisions on changes to medication regimens.		
108. van der Meer et al 2018	Randomized controlled trial	Community-dwelling patients aged ≥65 years who used ≥5 medicines for ≥3 months, including at least one psycholeptic/psychoanaleptic medication and who had a Drug Burden Index (DBI) ≥1. 15 community pharmacies in the Northern Netherlands.	Implicit.<br>Medication review by the community pharmacist:<br>1. in person consult: pharmacist and pt.<br>2. pharmacist identifies potential DRPs and and drafts recommendations.<br>3. meeting between pharmacist and patient, action plan was decided.<br>4. discussion of plan with patient &/or GP.<br>5. follow-up with patient.	Mortality, Hospitalizations, Falls, PIMs	3 months
109. Varas-Doval et al 2020	Cluster randomized controlled trial at practice level	Patients 64 + using 5 or more medicines. From community pharmacies in 4 provinces in Spain.	Implicit.<br>Medication review with follow-up using the Dader method:<br>1. analysis of pts medication therapy: interview with pt to assess pharmacotherapy<br>2. care plan: interventions directed to the physician or to the patient to improve DRPs<br>3. follow-up: monthly visits to pts for assessment of interventions' results and continuance with patients' care plan.	Hospitalizations	6 months
110. Verdoorn et al 2019	Randomized controlled trial	Community-dwelling persons aged 70 + taking more than 7 long-term medications, from general practice clinics and 35 community pharmacies in Netherlands.	Implicit.<br>Clinical medication review:<br>1. interview by pharmacist to identify problems and propose health-related goals.<br>2. summary of the drug related problems and recommendations.	Hospitalizations, ED visits, Quality of Life, Medical visits, Number of medications	6 months

© 2025 Persaud N et al. *JAMA Network Open*.

			3.meeting with patient's GP to discuss plan.<br>4. discussion with patient to agree on plan.<br>5. follow-up to evaluate progress/ adjust.		
111. Vicens et al 2022	Cluster randomized controlled trial at facility level	Patients at primary care centres in 3 different regions of Spain	Implicit.<br>GPs from the intervention group received a 2-hour educational workshop. After the initial training and workshop, GPs received automated monthly feedback to provide a summary of their clinical performance.	PIMs	12 months
112. Wallis et al 2021	Cluster randomized controlled trial at practice level.	Patients at increased risk of gastrointestinal, renal, or cardiac ADEs from NSAIDs or anti-platelet medications, from general practice clinics in New Zealand.	Explicit.<br>Safer Prescribing and Care for the Elderly (SPACE) intervention<br>1. Automated search of practice records to identify patients with high risk of ADEs related to NSAIDs or antiplatelet medications.<br>2. 1-hour educational sessions for GPs led by clinical advisory pharmacist on appropriate prescribing practices of NSAIDs and anti-platelet medications.<br>3. One-on-One Meetings with GPs and pharmacists to determine intended action for each patient.<br>4. GPs send letters to selected pts, prompting them to discuss their medicines in next appointment.	Hospitalizations, PIMs	12 months
113. Weber et al 2008	Cluster randomized controlled trial at practice level	Community-dwelling patients aged 70+, at risk for falls based on age and medication use, in US.	Explicit.<br>Computerized software tool: standardized medication review by clinical pharmacist or geriatric pharmacology expert via EMR. Following review, a message was sent to physician with specific recommendations (Fall prevention guideline based on the American Geriatrics Society/American Academy of Orthopedic Surgery fall prevention guidelines)	Mortality, Falls, Number of medications	15 months
114. Willeboordse et al 2017	Cluster randomized	Patients of ≥ 65 years if they newly presented with pre-specified geriatric symptoms	Explicit.<br>Clinical Medical Reviews (CMR).<br>Preparation: Gather information from		6 months

© 2025 Persaud N et al. *JAMA Network Open*.

	controlled trial by practice	and had the chronic use of ≥ 1 prescribed drug, from 22 general practice clinics in Netherlands.	EMRs, pharmacy records, and screening. Discussed with the patient by the GP.		
115. Williams et al 2004	Randomized controlled trial	Patients aged 65 +, cognitively intact, taking a minimum of 5 prescription medications. At least 2 of the medications were potentially problematic drugs for common geriatric problems, from ambulatory clinics from the General Medicine Clinic of the University of North Carolina Hospital's patient data pool and from private practices in the Chapel Hill area in US.	Implicit.<br>Medication Review: Comprehensive review with a consultant pharmacist, discussion with patient and GP and recommended modification of a patient's medication regimen.	Number of medications	1.5 months
116. Wouters et al 2017	Cluster randomized controlled trial at provider level.	Nursing home residents with a life expectancy > 4 weeks who consented to treatment with medication, in 59 wards for long-term care in Netherlands.	Explicit.<br>Multistep Medication Review: assessment of the patient perspective, medical history, critical appraisal of medications, a meeting between the treating elder care physician and the pharmacist, and implementation of medication changes.	Quality of Life, Falls, Medical visits, PIMs	4 months
117. Zechmann et al 2020	Cluster randomized controlled trial at provider level.	Patients ≥ age 60, taking ≥ 5 drugs for ≥ 6 months, from 46 primary care physicians in Switzerland.	Implicit.<br>PCP's: 2-hour training encouraging the use of a deprescribing-algorithm adapted from the Good-Palliative-Geriatric Practice algorithm and training on Shared Decision-Making principles. Patient-level: PN or PCP obtained list of current patient medications, evaluated appropriateness and made recommendations.	Mortality, Hospitalizations, Quality of Life, Number of medications	12 months
118. Zermansky et al 2001	Randomized controlled trial	Patients ≥ age 65, taking at least 2 repeat prescriptions, from 4 general practice clinics in UK.	Implicit.<br>Medication review where patients were invited to a consultation at which the pharmacist reviewed their medical conditions and current treatment in 3 stages: (1) Data gathering - Patient	Mortality, Hospitalizations, Medical visits, Number of medications	12 months

© 2025 Persaud N et al. *JAMA Network Open*.

			interview to record current drugs and active medical problems; (2) Evaluation - Review active medication use, side-effects, drug interactions, contraindications, cost; (3) Implementation – Determine if patient needs major changes, minor changes, or no changes to their prescription.		

DBI: drug burden index, DRP: drug-related problem, ED: emergency department, EMR: electronic medical record, GP: general practitioner, MAI: medication appropriateness index, MD: medical doctor, N°: number, PCI: pharmaceutical care issues, PIM: potentially inappropriate medication, PIP: potentially inappropriate prescribing, PPO: potentially prescribing omission, PT(s): patient(s), RN: registered nurse.

© 2025 Persaud N et al. *JAMA Network Open*.

eReferences

1. Allard J, Hébert R, Rioux M, et al. Efficacy of a clinical medication review on the number of potentially inappropriate prescriptions prescribed for community-dwelling elderly people. *CMAJ.* 2001;164(9):1291-1296.

2. Almutairi H, Stafford A, Etherton-Beer C, et al. Impact of a Multifaceted, Pharmacist-Led Intervention on Psychotropic Medication Use for Residents of Aged Care Facilities: A Parallel Cluster Randomized Controlled Trial. J Am Med Dir Assoc. 2023 Sep;24(9):1311.e1-1311.e8. doi: 10.1016/j.jamda.2023.06.037. Epub 2023 Aug 8. PMID: 37567242.

3. Amorim, W.W., Passos, L.C., Gama, R.S. et al. Using a mobile application to reduce potentially inappropriate prescribing for older Brazilian adults in primary care: a triple-blind randomised clinical trial. BMC Geriatr 24, 35 (2024). https://doi.org/10.1186/s12877-023-04645-z

4. Auvinen KJ, Räisänen J, Voutilainen A, et al. Interprofessional Medication Assessment has Effects on the Quality of Medication Among Home Care Patients: Randomized Controlled Intervention Study. *J Am Med Dir Assoc*. 2021;22(1):74-81. doi:10.1016/j.jamda.2020.07.007

5. Auvinen K, Voutilainen A, Jyrkkä J, et al. Interprofessional medication assessment among home care patients: any impact on functioning? Results from a randomised controlled trial. *BMC Geriatr*. 2020;20(1):390. Published 2020 Oct 6. doi:10.1186/s12877-020-01796-1

6. Avorn J, Soumerai SB, Everitt DE, et al. A randomized trial of a program to reduce the use of psychoactive drugs in nursing homes. *N Engl J Med*. 1992;327(3):168-173. doi:10.1056/NEJM199207163270306

7. Balsom C, Pittman N, King R, et al. Impact of a pharmacist-administered deprescribing intervention on nursing home residents: a randomized controlled trial. *Int J Clin Pharm*. 2020;42(4):1153-1167. doi:10.1007/s11096-020-01073-6

8. Basger BJ, Moles RJ, Chen TF. Impact of an enhanced pharmacy discharge service on prescribing appropriateness criteria: a randomised controlled trial. *Int J Clin Pharm*. 2015;37(6):1194-1205. doi:10.1007/s11096-015-0186-0

9. Bayliss EA, Shetterly SM, Drace ML, et al. Deprescribing Education vs Usual Care for Patients With Cognitive Impairment and Primary Care Clinicians: The OPTIMIZE Pragmatic Cluster Randomized Trial. *JAMA Intern Med*. 2022;182(5):534-542. doi:10.1001/jamainternmed.2022.0502

10. Beer C, Loh PK, Peng YG, et al. A pilot randomized controlled trial of deprescribing. *Ther Adv Drug Saf*. 2011;2(2):37-43. doi:10.1177/2042098611400332

11. Bogaerts JMK, Gussekloo J, de Jong-Schmit BEM, et al. Effects of the discontinuation of antihypertensive treatment on neuropsychiatric symptoms and quality of life in nursing home residents with dementia (DANTON): a multicentre, open-label, blinded-outcome, randomised controlled trial. Age Ageing. 2024 Jul 2;53(7):afae133. doi: 10.1093/ageing/afae133. PMID: 38970547; PMCID: PMC11227112.

12. Bosch-Lenders D, Jansen J, Stoffers HEJHJ, et al. The Effect of a Comprehensive, Interdisciplinary Medication Review on Quality of Life and Medication Use in Community Dwelling Older People with Polypharmacy. *J Clin Med*. 2021;10(4):600. Published 2021 Feb 5. doi:10.3390/jcm10040600

13. Boyd CM, Shetterly SM, Powers JD, et al. Evaluating the Safety of an Educational Deprescribing Intervention: Lessons from the Optimize Trial. Drugs Aging. 2024 Jan;41(1):45-54. doi: 10.1007/s40266-023-01080-y. Epub 2023 Nov 20. PMID: 37982982; PMCID: PMC11101016.

© 2025 Persaud N et al. *JAMA Network Open*.

14. Bregnhøj L, Thirstrup S, Kristensen MB, et al. Combined intervention programme reduces inappropriate prescribing in elderly patients exposed to polypharmacy in primary care. *Eur J Clin Pharmacol*. 2009;65(2):199-207. doi:10.1007/s00228-008-0558-7

15. Briggs S, Pearce R, Dilworth S, et al. Clinical pharmacist review: a randomised controlled trial. *Emerg Med Australas*. 2015;27(5):419-426. doi:10.1111/1742-6723.12451

16. Brünn R, Basten J, Lemke D, et al. Digital Medication Management in Polypharmacy. Dtsch Arztebl Int. 2024 Apr 19;121(8):243-250. doi: 10.3238/arztebl.m2024.0007. PMID: 38377330; PMCID: PMC11381212.

17. Bryant LJ, Coster G, Gamble GD, et al. The General Practitioner-Pharmacist Collaboration (GPPC) study: a randomised controlled trial of clinical medication reviews in community pharmacy. *Int J Pharm Pract*. 2011;19(2):94-105. doi:10.1111/j.2042-7174.2010.00079.x

18. Callegari E, Benth JŠ, Selbæk G, et al. The Effect of the NorGeP-NH on Quality of Life and Drug Prescriptions in Norwegian Nursing Homes: A Randomized Controlled Trial. Pharmacy (Basel). 2022 Feb 16;10(1):32. doi: 10.3390/pharmacy10010032. PMID: 35202081; PMCID: PMC8880047.

19. Campbell NL, Holden RJ, Tang Q, et al. Multicomponent behavioral intervention to reduce exposure to anticholinergics in primary care older adults. *J Am Geriatr Soc.* 2021;69(6):1490-1499. doi:10.1111/jgs.17121

20. Campins L, Serra-Prat M, Gózalo I, et al. Randomized controlled trial of an intervention to improve drug appropriateness in community-dwelling polymedicated elderly people. *Fam Pract.* 2017;34(1):36-42. doi:10.1093/fampra/cmw073

21. Cateau D, Ballabeni P, Niquille A. Effects of an interprofessional deprescribing intervention in Swiss nursing homes: the Individual Deprescribing Intervention (IDeI) randomised controlled trial. *BMC Geriatr*. 2021;21(1):655. Published 2021 Nov 19. doi:10.1186/s12877-021-02465-7

22. Cateau D, Ballabeni P, Niquille A. Effects of an interprofessional Quality Circle-Deprescribing Module (QC-DeMo) in Swiss nursing homes: a randomised controlled trial. *BMC Geriatr*. 2021;21(1):289. Published 2021 May 1. doi:10.1186/s12877-021-02220-y

23. Clyne B, Smith SM, Hughes CM, et al. Effectiveness of a Multifaceted Intervention for Potentially Inappropriate Prescribing in Older Patients in Primary Care: A Cluster-Randomized Controlled Trial (OPTI-SCRIPT Study). *Ann Fam Med.* 2015;13(6):545-553. doi:10.1370/afm.1838

24. Clyne B, Smith SM, Hughes CM, et al. Sustained effectiveness of a multifaceted intervention to reduce potentially inappropriate prescribing in older patients in primary care (OPTI-SCRIPT study). *Implement Sci*. 2016;11(1):79. Published 2016 Jun 2. doi:10.1186/s13012-016-0442-2

25. Coronado-Vázquez V, Gómez-Salgado J, Cerezo-Espinosa de Los Monteros J, et al. Shared Decision-Making in Chronic Patients with Polypharmacy: An Interventional Study for Assessing Medication Appropriateness. *J Clin Med.* 2019;8(6):904. Published 2019 Jun 24. doi:10.3390/jcm8060904

26. Crotty M, Halbert J, Rowett D, et al. An outreach geriatric medication advisory service in residential aged care: a randomised controlled trial of case conferencing. *Age Ageing.* 2004;33(6):612-617. doi:10.1093/ageing/afh213

27. Del Cura-González I, López-Rodríguez JA, Leiva-Fernández F, et al. How to Improve Healthcare for Patients with Multimorbidity and Polypharmacy in Primary Care: A Pragmatic Cluster-Randomized

© 2025 Persaud N et al. *JAMA Network Open*.

Clinical Trial of the MULTIPAP Intervention. *J Pers Med*. 2022;12(5):752. Published 2022 May 6. doi:10.3390/jpm12050752

28. Desborough JA, Clark A, Houghton J, et al. Clinical and cost effectiveness of a multi-professional medication reviews in care homes (CAREMED). *Int J Pharm Pract.* 2020;28(6):626-634. doi:10.1111/ijpp.12656

29. Dugré N, Bell JS, Hopkins RE, et al. Impact of Medication Regimen Simplification on Medication Incidents in Residential Aged Care: SIMPLER Randomized Controlled Trial. *J Clin Med.* 2021;10(5):1104. Published 2021 Mar 6. doi:10.3390/jcm10051104

30. Elliott LS, Henderson JC, Neradilek MB, et al. Clinical impact of pharmacogenetic profiling with a clinical decision support tool in polypharmacy home health patients: A prospective pilot randomized controlled trial. *PLoS One.* 2017;12(2):e0170905. Published 2017 Feb 2. doi:10.1371/journal.pone.0170905

31. Erler A, Beyer M, Petersen JJ, et al. How to improve drug dosing for patients with renal impairment in primary care - a cluster-randomized controlled trial. *BMC Fam Pract*. 2012;13:91. Published 2012 Sep 6. doi:10.1186/1471-2296-13-91

32. Etherton-Beer C, Page A, Naganathan V, et al. Deprescribing to optimise health outcomes for frail older people: a double-blind placebo-controlled randomised controlled trial-outcomes of the Opti-med study. Age Ageing. 2023 May 1;52(5):afad081. doi: 10.1093/ageing/afad081. PMID: 37247404; PMCID: PMC10226731.

33. Fournier A, Anrys P, Beuscart JB, et al. Use and Deprescribing of Potentially Inappropriate Medications in Frail Nursing Home Residents. *Drugs Aging.* 2020;37(12):917-924. doi:10.1007/s40266-020-00805-7

34. Frankenthal D, Lerman Y, Kalendaryev E, et al. Intervention with the screening tool of older persons potentially inappropriate prescriptions/screening tool to alert doctors to right treatment criteria in elderly residents of a chronic geriatric facility: a randomized clinical trial. *J Am Geriatr Soc.* 2014;62(9):1658-1665. doi:10.1111/jgs.12993

35. Fried TR, Niehoff KM, Street RL, et al. Effect of the Tool to Reduce Inappropriate Medications on Medication Communication and Deprescribing. *J Am Geriatr Soc*. 2017;65(10):2265-2271. doi:10.1111/jgs.15042

36. Furniss L, Burns A, Craig SK, et al. Effects of a pharmacist's medication review in nursing homes. Randomised controlled trial. *Br J Psychiatry*. 2000;176:563-567. doi:10.1192/bjp.176.6.563

37. García-Gollarte F, Baleriola-Júlvez J, Ferrero-López I, et al. An educational intervention on drug use in nursing homes improves health outcomes resource utilization and reduces inappropriate drug prescription. *J Am Med Dir Assoc.* 2014;15(12):885-891. doi:10.1016/j.jamda.2014.04.010

38. Gedde MH, Husebo BS, Mannseth J, et al. Less Is More: The Impact of Deprescribing Psychotropic Drugs on Behavioral and Psychological Symptoms and Daily Functioning in Nursing Home Patients. Results From the Cluster-Randomized Controlled COSMOS Trial. *Am J Geriatr Psychiatry*. 2021;29(3):304-315. doi:10.1016/j.jagp.2020.07.004

39. Gedde MH, Husebo BS, Mannseth J, et al. The impact of medication reviews by general practitioners on psychotropic drug use and behavioral and psychological symptoms in home-dwelling people with dementia: results from the multicomponent cluster randomized controlled LIVE@Home.Path trial. *BMC Med*. 2022;20(1):186. doi:10.1186/s12916-022-02382-5

© 2025 Persaud N et al. *JAMA Network Open*.

40. Green AR, Quiles R, Daddato AE, et al. Pharmacist-led telehealth deprescribing for people living with dementia and polypharmacy in primary care: A pilot study. *J Am Geriatr Soc*. 2024;72(7):1973-1984. doi:10.1111/jgs.18867

41. Geurts MM, Stewart RE, Brouwers JR, et al. Implications of a clinical medication review and a pharmaceutical care plan of polypharmacy patients with a cardiovascular disorder. *Int J Clin Pharm.* 2016;38(4):808-815. doi:10.1007/s11096-016-0281-x

42. Haag JD, Davis AZ, Hoel RW, et al. Impact of Pharmacist-Provided Medication Therapy Management on Healthcare Quality and Utilization in Recently Discharged Elderly Patients. *Am Health Drug Benefits.* 2016;9(5):259-268.

43. Haider I, Kosari S, Naunton M, et al. Impact of on-site pharmacists in residential aged care facilities on the quality of medicines use: a cluster randomised controlled trial (PiRACF study). *Sci Rep*. 2023;13(1):15962. doi:10.1038/s41598-023-42894-5

44. Hanlon JT, Weinberger M, Samsa GP, et al. A randomized, controlled trial of a clinical pharmacist intervention to improve inappropriate prescribing in elderly outpatients with polypharmacy. *Am J Med*. 1996;100(4):428-437. doi:10.1016/S0002-9343(97)89519-8

45. Harnisch M, Barnett ML, Coussens S, et al. Physician Antipsychotic Overprescribing Letters and Cognitive, Behavioral, and Physical Health Outcomes Among People With Dementia: A Secondary Analysis of a Randomized Clinical Trial. *JAMA Netw Open*. 2024;7(4):e247604. doi:10.1001/jamanetworkopen.2024.7604

46. Herrinton LJ, Lo K, Alavi M, et al. Effectiveness of Bundled Hyperpolypharmacy Deprescribing Compared With Usual Care Among Older Adults: A Randomized Clinical Trial. *JAMA Netw Open*. 2023;6(7):e2322505. Published 2023 Jul 3. doi:10.1001/jamanetworkopen.2023.22505

47. Holdhus H, Bøvre K, Mathiesen L, Bjelke B, Bjerknes K. Limited effect of structured medication report as the only intervention at discharge from hospital. *Eur J Hosp Pharm*. 2019;26(2):101-105. doi:10.1136/ejhpharm-2017-001371

48. Holland R, Bond C, Alldred DP, et al. Evaluation of effectiveness and safety of pharmacist independent prescribers in care homes: cluster randomised controlled trial [published correction appears in BMJ. 2023 Feb 23;380:p446. doi: 10.1136/bmj.p446.]. BMJ. 2023;380:e071883. Published 2023 Feb 14. doi:10.1136/bmj-2022-071883

49. Johansson KS, Kornholt J, Bülow C, et al. Physician-led medication reviews in polypharmacy patients treated with at least 12 medications in a type 2 diabetes outpatient clinic: A randomised trial. Diabet Med. 2023;40(4):e15052. doi:10.1111/dme.15052

50. Jungo KT, Ansorg AK, Floriani C, et al. Optimising prescribing in older adults with multimorbidity and polypharmacy in primary care (OPTICA): cluster randomised clinical trial. BMJ. 2023;381:e074054. Published 2023 May 24. doi:10.1136/bmj-2022-074054

51. Junius-Walker U, Krause O, Thürmann P, et al. Drug Safety for Nursing-Home Residents-Findings of a Pragmatic, Cluster-Randomized, Controlled Intervention Trialin 44 Nursing Homes. *Dtsch Arztebl Int.* 2021;118(42):705-712. doi:10.3238/arztebl.m2021.0297

52. Juola AL, Bjorkman MP, Pylkkanen S, et al. Nurse Education to Reduce Harmful Medication Use in Assisted Living Facilities: Effects of a Randomized Controlled Trial on Falls and Cognition. *Drugs Aging*. 2015;32(11):947-955. doi:10.1007/s40266-015-0311-8

© 2025 Persaud N et al. *JAMA Network Open*.

53. Kirkham J, Maxwell C, Velkers C, Leung R, Moffat K, Seitz D. Optimizing Prescribing of Antipsychotics in Long-Term Care (OPAL): A Stepped-Wedge Trial. *J Am Med Dir Assoc*. 2020;21(3):381-387.e3. doi:10.1016/j.jamda.2019.07.025

54. Köberlein-Neu J, Mennemann H, Hamacher S, et al. Interprofessional Medication Management in Patients With Multiple Morbidities. *Dtsch Arztebl Int*. 2016;113(44):741-748. doi:10.3238/arztebl.2016.0741

55. Könner F, Budnick A, Kuhnert R, et al. Interventions to address deficits of pharmacological pain management in nursing home residents--A cluster-randomized trial. *Eur J Pain*. 2015;19(9):1331-1341. doi:10.1002/ejp.663

56. Kornholt J, Feizi ST, Hansen AS, et al. Effects of a comprehensive medication review intervention on health-related quality of life and other clinical outcomes in geriatric outpatients with polypharmacy: A pragmatic randomized clinical trial. *Br J Clin Pharmacol*. 2022;88(7):3360-3369. doi:10.1111/bcp.15287

57. Kornholt J, Feizi ST, Hansen AS, et al. Medication changes implemented during medication reviews and factors related to deprescribing: Posthoc analyses of a randomized clinical trial in geriatric outpatients with polypharmacy. Br J Clin Pharmacol. 2023;89(11):3291-3301. doi:10.1111/bcp.15805

58. Kouladjian O'Donnell L, Gnjidic D, Sawan M, et al. Impact of the Goal-directed Medication Review Electronic Decision Support System on Drug Burden Index: A cluster-randomised clinical trial in primary care. *Br J Clin Pharmacol*. 2021;87(3):1499-1511. doi:10.1111/bcp.14557

59. Krska J, Cromarty JA, Arris F, et al. Pharmacist-led medication review in patients over 65: a randomized, controlled trial in primary care. *Age Ageing*. 2001;30(3):205-211. doi:10.1093/ageing/30.3.205

60. Kua CH, Yeo CYY, Tan PC, et al. Association of Deprescribing With Reduction in Mortality and Hospitalization: A Pragmatic Stepped-Wedge Cluster-Randomized Controlled Trial. *J Am Med Dir Assoc*. 2021;22(1):82-89.e3. doi:10.1016/j.jamda.2020.03.012

61. Kwint HF, Faber A, Gussekloo J, Bouvy ML. Effects of medication review on drug-related problems in patients using automated drug-dispensing systems: a pragmatic randomized controlled study. *Drugs Aging*. 2011;28(4):305-314. doi:10.2165/11586850-000000000-00000

62. Lampela P, Hartikainen S, Lavikainen P, Sulkava R, Huupponen R. Effects of medication assessment as part of a comprehensive geriatric assessment on drug use over a 1-year period: a population-based intervention study. *Drugs Aging*. 2010;27(6):507-521. doi:10.2165/11536650-000000000-00000

63. Lenaghan E, Holland R, Brooks A. Home-based medication review in a high risk elderly population in primary care--the POLYMED randomised controlled trial. *Age Ageing*. 2007;36(3):292-297. doi:10.1093/ageing/afm036

64. Lenander, C.,Elfsson, B.,Danielsson, B.,Midlov, P.,Hasselstrom, J. (2014). Effects of a pharmacist-led structured medication review in primary care on drug-related problems and hospital admission rates: a randomized controlled trial Scandinavian Journal of Primary Health Care, 32(4), 180-6

65. Liu AK, Possin KL, Cook KM, et al. Effect of collaborative dementia care on potentially inappropriate medication use: Outcomes from the Care Ecosystem randomized clinical trial. *Alzheimers Dement*. 2023;19(5):1865-1875. doi:10.1002/alz.12808

66. Lin HW, Lin CH, Chang CK, et al. Economic outcomes of pharmacist-physician medication therapy management for polypharmacy elderly: A prospective, randomized, controlled trial. *J Formos Med Assoc*. 2018;117(3):235-243. doi:10.1016/j.jfma.2017.04.017

© 2025 Persaud N et al. *JAMA Network Open*.

67. Liou WS, Huang SM, Lee WH, Chang YL, Wu MF. The effects of a pharmacist-led medication review in a nursing home: A randomized controlled trial. *Medicine (Baltimore)*. 2021;100(48):e28023. doi:10.1097/MD.0000000000028023

68. Liu AK, Possin KL, Cook KM, et al. Effect of collaborative dementia care on potentially inappropriate medication use: Outcomes from the Care Ecosystem randomized clinical trial. Alzheimers Dement. 2023;19(5):1865-1875. doi:10.1002/alz.12808

69. Mahlknecht A, Wiedermann CJ, Sandri M, et al. Expert-based medication reviews to reduce polypharmacy in older patients in primary care: a northern-Italian cluster-randomised controlled trial. *BMC Geriatr*. 2021;21(1):659. Published 2021 Nov 23. doi:10.1186/s12877-021-02612-0

70. Malet-Larrea A, Goyenechea E, García-Cárdenas V, et al. The impact of a medication review with follow-up service on hospital admissions in aged polypharmacy patients. *Br J Clin Pharmacol*. 2016;82(3):831-838. doi:10.1111/bcp.13012

71. Martin P, Tamblyn R, Benedetti A, Ahmed S, Tannenbaum C. Effect of a Pharmacist-Led Educational Intervention on Inappropriate Medication Prescriptions in Older Adults: The D-PRESCRIBE Randomized Clinical Trial. *JAMA*. 2018;320(18):1889-1898. doi:10.1001/jama.2018.16131

72. McCarthy C, Clyne B, Boland F, et al. GP-delivered medication review of polypharmacy, deprescribing, and patient priorities in older people with multimorbidity in Irish primary care (SPPiRE Study): A cluster randomised controlled trial. *PLoS Med*. 2022;19(1):e1003862. Published 2022 Jan 5. doi:10.1371/journal.pmed.1003862

73. Milos V, Rekman E, Bondesson Å, et al. Improving the quality of pharmacotherapy in elderly primary care patients through medication reviews: a randomised controlled study. *Drugs Aging*. 2013;30(4):235-246. doi:10.1007/s40266-013-0057-0

74. Milos Nymberg V, Lenander C, Borgström Bolmsjö B. The Impact of Medication Reviews Conducted in Primary Care on Hospital Admissions and Mortality: An Observational Follow-Up of a Randomized Controlled Trial. Drug Healthc Patient Saf. 2021;13:1-9. Published 2021 Jan 27. doi:10.2147/DHPS.S283708

75. Moga DC, Abner EL, Rigsby DN, et al. Optimizing medication appropriateness in older adults: a randomized clinical interventional trial to decrease anticholinergic burden. *Alzheimers Res Ther*. 2017;9(1):36. Published 2017 May 23. doi:10.1186/s13195-017-0263-9

76. Mortsiefer A, Löscher S, Pashutina Y, et al. Family Conferences to Facilitate Deprescribing in Older Outpatients With Frailty and With Polypharmacy: The COFRAIL Cluster Randomized Trial. JAMA Netw Open. 2023;6(3):e234723. Published 2023 Mar 1. doi:10.1001/jamanetworkopen.2023.4723

77. Muth C, Uhlmann L, Haefeli WE, et al. Effectiveness of a complex intervention on Prioritising Multimedication in Multimorbidity (PRIMUM) in primary care: results of a pragmatic cluster randomised controlled trial. *BMJ Open*. 2018;8(2):e017740. Published 2018 Feb 24. doi:10.1136/bmjopen-2017-017740

78. Navy HJ, Weffald L, Delate T, Patel RJ, Dugan JP. Clinical Pharmacist Intervention to Engage Older Adults in Reducing Use of Alprazolam. *Consult Pharm*. 2018;33(12):711-722. doi:10.4140/TCP.n.2018.711.

79. Olsson IN, Runnamo R, Engfeldt P. Drug treatment in the elderly: an intervention in primary care to enhance prescription quality and quality of life. *Scand J Prim Health Care*. 2012;30(1):3-9. doi:10.3109/02813432.2011.629149

© 2025 Persaud N et al. *JAMA Network Open*.

80. Parker K, Bull-Engelstad I, Benth JŠ, et al. Effectiveness of using STOPP/START criteria to identify potentially inappropriate medication in people aged ≥ 65 years with chronic kidney disease: a randomized clinical trial. *Eur J Clin Pharmacol*. 2019;75(11):1503-1511. doi:10.1007/s00228-019-02727-9

81. Patterson SM, Hughes CM, Crealey G, Cardwell C, Lapane KL. An evaluation of an adapted U.S. model of pharmaceutical care to improve psychoactive prescribing for nursing home residents in northern ireland (fleetwood northern ireland study). *J Am Geriatr Soc*. 2010;58(1):44-53. doi:10.1111/j.1532-5415.2009.02617.x

82. Phelan EA, Williamson BD, Balderson BH, et al. Reducing Central Nervous System-Active Medications to Prevent Falls and Injuries Among Older Adults: A Cluster Randomized Clinical Trial. *JAMA Netw Open*. 2024;7(7):e2424234. Published 2024 Jul 1. doi:10.1001/jamanetworkopen.2024.24234

83. Pitkälä KH, Juola AL, Kautiainen H, et al. Education to reduce potentially harmful medication use among residents of assisted living facilities: a randomized controlled trial. *J Am Med Dir Assoc*. 2014;15(12):892-898. doi:10.1016/j.jamda.2014.04.002

84. Pitkälä KH, Strandberg TE, Tilvis RS. Is it possible to reduce polypharmacy in the elderly? A randomised, controlled trial. *Drugs Aging*. 2001;18(2):143-149. doi:10.2165/00002512-200118020-00007

85. Potter K, Flicker L, Page A, Etherton-Beer C. Deprescribing in Frail Older People: A Randomised Controlled Trial. *PLoS One*. 2016;11(3):e0149984. Published 2016 Mar 4. doi:10.1371/journal.pone.0149984

86. Raebel MA, Charles J, Dugan J, et al. Randomized trial to improve prescribing safety in ambulatory elderly patients. *J Am Geriatr Soc*. 2007;55(7):977-985. doi:10.1111/j.1532-5415.2007.01202.x

87. Rankin A, Gorman A, Cole J, et al. An external pilot cluster randomised controlled trial of a theory-based intervention to improve appropriate polypharmacy in older people in primary care (PolyPrime). Pilot Feasibility Stud. 2022;8(1):203. Published 2022 Sep 10. doi:10.1186/s40814-022-01161-6

88. Richter C, Berg A, Langner H, et al. Effect of person-centred care on antipsychotic drug use in nursing homes (EPCentCare): a cluster-randomised controlled trial. *Age Ageing*. 2019;48(3):419-425. doi:10.1093/ageing/afz016

89. Rieckert A, Reeves D, Altiner A, et al. Use of an electronic decision support tool to reduce polypharmacy in elderly people with chronic diseases: cluster randomised controlled trial. *BMJ*. 2020;369:m1822. Published 2020 Jun 18. doi:10.1136/bmj.m1822

90. Rognstad S, Brekke M, Fetveit A, Dalen I, Straand J. Prescription peer academic detailing to reduce inappropriate prescribing for older patients: a cluster randomised controlled trial. *Br J Gen Pract*. 2013;63(613):e554-e562. doi:10.3399/bjgp13X670688

91. Romskaug R, Skovlund E, Straand J, et al. Effect of Clinical Geriatric Assessments and Collaborative Medication Reviews by Geriatrician and Family Physician for Improving Health-Related Quality of Life in Home-Dwelling Older Patients Receiving Polypharmacy: A Cluster Randomized Clinical Trial. *JAMA Intern Med*. 2020;180(2):181-189. doi:10.1001/jamainternmed.2019.5096

92. Roughead EE, Pratt NL, Parfitt G, et al. Effect of an ongoing pharmacist service to reduce medicine-induced deterioration and adverse reactions in aged-care facilities (nursing homes): a multicentre, randomised controlled trial (the ReMInDAR trial). Age Ageing. 2022;51(4):afac092. doi:10.1093/ageing/afac092

© 2025 Persaud N et al. *JAMA Network Open*.

93. Rudolf H, Thiem U, Aust K, et al. Reduction of Potentially Inappropriate Medication in the Elderly. *Dtsch Arztebl Int*. 2021;118(51-52):875-882. doi:10.3238/arztebl.m2021.0372

94. Sacarny A, Barnett ML, Le J, Tetkoski F, Yokum D, Agrawal S. Effect of Peer Comparison Letters for High-Volume Primary Care Prescribers of Quetiapine in Older and Disabled Adults: A Randomized Clinical Trial. *JAMA Psychiatry*. 2018;75(10):1003-1011. doi:10.1001/jamapsychiatry.2018.1867

95. Schäfer I, Kaduszkiewicz H, Mellert C, et al. Narrative medicine-based intervention in primary care to reduce polypharmacy: results from the cluster-randomised controlled trial MultiCare AGENDA. *BMJ Open*. 2018;8(1):e017653. Published 2018 Jan 23. doi:10.1136/bmjopen-2017-017653

96. Schmidt I, Claesson CB, Westerholm B, Nilsson LG, Svarstad BL. The impact of regular multidisciplinary team interventions on psychotropic prescribing in Swedish nursing homes. *J Am Geriatr Soc*. 1998;46(1):77-82. doi:10.1111/j.1532-5415.1998.tb01017.x

97. Schmidt-Mende K, Andersen M, Wettermark B, Hasselström J. Educational intervention on medication reviews aiming to reduce acute healthcare consumption in elderly patients with potentially inappropriate medicines-A pragmatic open-label cluster-randomized controlled trial in primary care. *Pharmacoepidemiol Drug Saf*. 2017;26(11):1347-1356. doi:10.1002/pds.4263

98. Sellors J, Kaczorowski J, Sellors C, et al. A randomized controlled trial of a pharmacist consultation program for family physicians and their elderly patients. *CMAJ*. 2003;169(1):17-22.

99. Sheppard JP, Temple E, Wang A, et al. Effect of antihypertensive deprescribing on hospitalisation and mortality: long-term follow-up of the OPTiMISE randomised controlled trial. Lancet Healthy Longev. 2024;5(8):e563-e573. doi:10.1016/S2666-7568(24)00131-4

100. Strauven G, Anrys P, Vandael E, et al. Cluster-Controlled Trial of an Intervention to Improve Prescribing in Nursing Homes Study. *J Am Med Dir Assoc*. 2019;20(11):1404-1411. doi:10.1016/j.jamda.2019.06.006

101. Sultan R, van den Beukel TO, Reumerman MO, et al. An Interprofessional Student-Run Medication Review Program: The Clinical STOPP/START-Based Outcomes of a Controlled Clinical Trial in a Geriatric Outpatient Clinic. Clin Pharmacol Ther. 2022;111(4):931-938. doi:10.1002/cpt.2475

102. Syafhan NF, Al Azzam S, Williams SD, et al. General practitioner practice-based pharmacist input to medicines optimisation in the UK: pragmatic, multicenter, randomised, controlled trial. *J Pharm Policy Pract*. 2021;14(1):4. Published 2021 Jan 4. doi:10.1186/s40545-020-00279-3

103. Tadrous M, Fung K, Desveaux L, et al. Effect of Academic Detailing on Promoting Appropriate Prescribing of Antipsychotic Medication in Nursing Homes: A Cluster Randomized Clinical Trial. *JAMA Netw Open*. 2020;3(5):e205724. Published 2020 May 1. doi:10.1001/jamanetworkopen.2020.5724

104. Tamblyn R, Huang A, Perreault R, et al. The medical office of the 21st century (MOXXI): effectiveness of computerized decision-making support in reducing inappropriate prescribing in primary care. *CMAJ*. 2003;169(6):549-556.

105. Tannenbaum C, Martin P, Tamblyn R, Benedetti A, Ahmed S. Reduction of inappropriate benzodiazepine prescriptions among older adults through direct patient education: the EMPOWER cluster randomized trial. *JAMA Intern Med*. 2014;174(6):890-898. doi:10.1001/jamainternmed.2014.949

106. Terrell KM, Perkins AJ, Dexter PR, Hui SL, Callahan CM, Miller DK. Computerized decision support to reduce potentially inappropriate prescribing to older emergency department patients: a randomized, controlled trial. *J Am Geriatr Soc*. 2009;57(8):1388-1394. doi:10.1111/j.1532-5415.2009.02352.x

© 2025 Persaud N et al. *JAMA Network Open*.

107. Toivo T, Airaksinen M, Dimitrow M, et al. Enhanced coordination of care to reduce medication risks in older home care clients in primary care: a randomized controlled trial. *BMC Geriatr*. 2019;19(1):332. Published 2019 Nov 27. doi:10.1186/s12877-019-1353-2

108. van der Meer HG, Wouters H, Pont LG, Taxis K. Reducing the anticholinergic and sedative load in older patients on polypharmacy by pharmacist-led medication review: a randomised controlled trial. *BMJ Open*. 2018;8(7):e019042. Published 2018 Jul 19. doi:10.1136/bmjopen-2017-019042

109. Varas-Doval R, Gastelurrutia MA, Benrimoj SI, García-Cárdenas V, Sáez-Benito L, Martinez-Martínez F. Clinical impact of a pharmacist-led medication review with follow up for aged polypharmacy patients: A cluster randomized controlled trial. *Pharm Pract (Granada)*. 2020;18(4):2133. doi:10.18549/PharmPract.2020.4.2133

110. Verdoorn S, Kwint HF, Blom JW, Gussekloo J, Bouvy ML. Effects of a clinical medication review focused on personal goals, quality of life, and health problems in older persons with polypharmacy: A randomised controlled trial (DREAMeR-study). *PLoS Med*. 2019;16(5):e1002798. Published 2019 May 8. doi:10.1371/journal.pmed.1002798

111. Vicens C, Leiva A, Bejarano F, et al. Evaluation of a multicomponent intervention consisting of education and feedback to reduce benzodiazepine prescriptions by general practitioners: The BENZORED hybrid type 1 cluster randomized controlled trial. PLoS Med. 2022;19(5):e1003983. Published 2022 May 6. doi:10.1371/journal.pmed.1003983

112. Wallis KA, Elley CR, Moyes SA, Lee A, Hikaka JF, Kerse NM. Safer Prescribing and Care for the Elderly (SPACE): a cluster randomised controlled trial in general practice. *BJGP Open*. 2022;6(1):BJGPO.2021.0129. Published 2022 Mar 22. doi:10.3399/BJGPO.2021.0129

113. Weber V, White A, McIlvried R. An electronic medical record (EMR)-based intervention to reduce polypharmacy and falls in an ambulatory rural elderly population. *J Gen Intern Med*. 2008;23(4):399-404. doi:10.1007/s11606-007-0482-z

114. Willeboordse F, Schellevis FG, Chau SH, Hugtenburg JG, Elders PJM. The effectiveness of optimised clinical medication reviews for geriatric patients: Opti-Med a cluster randomised controlled trial. *Fam Pract*. 2017;34(4):437-445. doi:10.1093/fampra/cmx007

115. Williams ME, Pulliam CC, Hunter R, et al. The short-term effect of interdisciplinary medication review on function and cost in ambulatory elderly people. *J Am Geriatr Soc*. 2004;52(1):93-98. doi:10.1111/j.1532-5415.2004.52016.x

116. Wouters H, Scheper J, Koning H, et al. Discontinuing Inappropriate Medication Use in Nursing Home Residents: A Cluster Randomized Controlled Trial. *Ann Intern Med*. 2017;167(9):609-617. doi:10.7326/M16-2729

117. Zechmann S, Senn O, Valeri F, et al. Effect of a patient-centred deprescribing procedure in older multimorbid patients in Swiss primary care - A cluster-randomised clinical trial. *BMC Geriatr.* 2020;20(1):471. Published 2020 Nov 16. doi:10.1186/s12877-020-01870-8

118. Zermansky AG, Petty DR, Raynor DK, Freemantle N, Vail A, Lowe CJ. Randomised controlled trial of clinical medication review by a pharmacist of elderly patients receiving repeat prescriptions in general practice. *BMJ*. 2001;323(7325):1340-1343. doi:10.1136/bmj.323.7325.1340

© 2025 Persaud N et al. *JAMA Network Open*.

eTable 2. Examples of Excluded Studies

**Study**	**Reason for exclusion**
Andrade AQ, Calabretto JP, Pratt NL, et al. Implementation and Evaluation of a Digitally Enabled Precision Public Health Intervention to Reduce Inappropriate Gabapentinoid Prescription: Cluster Randomized Controlled Trial. *J Med Internet Res*. 2022;24(1):e33873. Published 2022 Jan 10. doi:10.2196/33873	Study aimed to evaluate intervention, not reduce PIMs.
Arnold SH, Nygaard Jensen J, Bjerrum L, et al. Effectiveness of a tailored intervention to reduce antibiotics for urinary tract infections in nursing home residents: a cluster, randomised controlled trial. *Lancet Infect Dis*. 2021;21(11):1549-1556. doi:10.1016/S1473-3099(21)00001-3	Antibiotic deprescribing focus.
Fletcher J, Hogg W, Farrell B, et al. Effect of nurse practitioner and pharmacist counseling on inappropriate medication use in family practice. *Can Fam Physician*. 2012;58(8):862-868.	Only includes patients in the intervention arm.
Gulliford MC, Prevost AT, Charlton J, et al. Effectiveness and safety of electronically delivered prescribing feedback and decision support on antibiotic use for respiratory illness in primary care: REDUCE cluster randomised trial. *BMJ*. 2019;364:l236. Published 2019 Feb 12. doi:10.1136/bmj.l236	Antibiotic deprescribing focus.
Hemkens LG, Saccilotto R, Reyes SL, et al. Personalized Prescription Feedback Using Routinely Collected Data to Reduce Antibiotic Use in Primary Care: A Randomized Clinical Trial. *JAMA Intern Med*. 2017;177(2):176-183. doi:10.1001/jamainternmed.2016.8040	Antibiotic deprescribing focus.
Holland R, Leneghan E, Smith R, et al. Delivering a home-based medication review, process measures from the HOMER randomised controlled trial. *Int J Pharm Pract.* 2006;14(1):71-79. doi:10.1211/ijpp.14.1.0009	Only includes patients in the intervention arm.
Nace DA, Hanlon JT, Crnich CJ, et al. A Multifaceted Antimicrobial Stewardship Program for the Treatment of Uncomplicated Cystitis in Nursing Home Residents. *JAMA Intern Med*. 2020;180(7):944-951. doi:10.1001/jamainternmed.2020.1256	Antibiotic deprescribing focus.
Pevnick JM, Nguyen C, Jackevicius CA, Palmer KA, Shane R, Cook-Wiens G, Rogatko A, Bear M, Rosen O, Seki D, Doyle B, Desai A, Bell DS. Improving admission medication reconciliation with pharmacists or pharmacy technicians in the emergency department: a randomised controlled trial. BMJ Qual Saf. 2018 Jul;27(7):512-520. doi: 10.1136/bmjqs-2017-006761. Epub 2017 Oct 6. PMID: 28986515; PMCID: PMC5912995.	Solely enrolled inpatients.
Rutten JJS, van Buul LW, Smalbrugge M, et al. An Electronic Health Record Integrated Decision Tool and Supportive Interventions to Improve Antibiotic Prescribing for Urinary Tract Infections in Nursing Homes: A Cluster Randomized Controlled Trial. *J Am Med Dir Assoc*. 2022;23(3):387-393. doi:10.1016/j.jamda.2021.11.010	Antibiotic deprescribing focus.
Schumacher PM, Griese-Mammen N, Schneider J, Laufs U, Schulz M. Interdisciplinary Physician-Pharmacist Medication Review for Outpatients With Heart Failure: A Subanalysis of the PHARM-CHF Randomized Controlled Trial. *Front Pharmacol*. 2021;12:712490. Published 2021 Sep 7. doi:10.3389/fphar.2021.712490	Study aimed to improve chronic health failure outcomes, not reduce PIMs.

eFigure 1. Summary of Risk of Bias Assessments for Included Studies

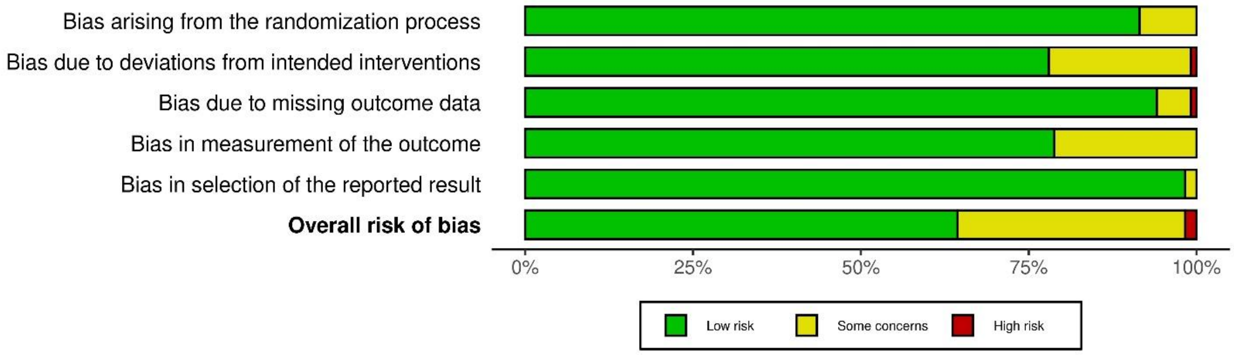

© 2025 Persaud N et al. *JAMA Network Open*.

eFigure 2. Risk of Bias Assessments for Each Included Trial

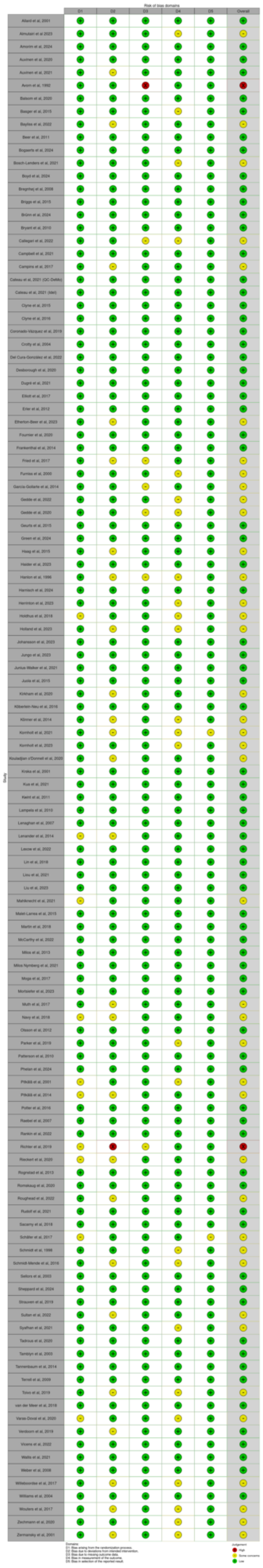

© 2025 Persaud N et al. *JAMA Network Open*.

eFigure 3. Nonserious Adverse Drug Reaction Effects of Potentially Inappropriate Prescribing Interventions

Study or Subgroup	Experimental Events	Experimental Total	Control Events	Control Total	Weight	Risk Ratio IV, Random, 95% CI
subgroup = implicit						
Del Cura−González et al. 2022	1	267	1	264	1.1%	0.99 [0.06; 15.73]
Hanlon et al. 1996	26	86	33	83	40.2%	0.76 [0.50; 1.15]
Total (95% CI)		**353**		**347**	**41.3%**	**0.76 [0.47; 1.25]**
Heterogeneity: $Tau^2 = 0$; $Chi^2 = 0.03$, df = 1 (P = 0.85); $I^2 = 0\%$						
subgroup = explicit						
Lenander et al. 2014	39	75	33	66	58.7%	1.04 [0.75; 1.44]
**Total (95% CI)**		**428**		**413**	**100.0%**	**0.92 [0.58; 1.46]**
Heterogeneity: $Tau^2 = 0.0110$; $Chi^2 = 1.35$, df = 2 (P = 0.51); $I^2 = 0\%$						
Test for subgroup differences: $Chi^2 = 3.26$, df = 1 (P = 0.07)						

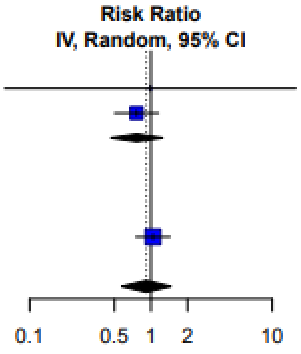

© 2025 Persaud N et al. *JAMA Network Open*.

eFigure 4. Falls Effects of Potentially Inappropriate Prescribing Interventions

Study or Subgroup	Experimental Mean	Experimental SD	Experimental Total	Control Mean	Control SD	Control Total	Weight	Std. Mean Difference IV, Random, 95% CI
subgroup = implicit								
Cateau et al. 2021 (Idel)	0.45	0.8900	27	0.48	0.8000	27	2.7%	−0.03 [−0.57; 0.50]
Desborough et al. 2020	3.35	8.3000	381	3.00	5.4900	445	5.6%	0.05 [−0.09; 0.19]
García−Gollarte et al. 2014	0.32	0.6800	344	0.43	0.8600	372	5.6%	−0.14 [−0.29; 0.01]
Junius−Walker et al. 2021	0.70	2.1000	452	0.50	1.6000	410	5.6%	0.11 [−0.03; 0.24]
Kornholt et al. 2022	0.27	0.1000	142	0.27	0.1000	150	5.0%	0.01 [−0.22; 0.24]
Kouladjian et al. 2021	0.07	0.5000	88	0.07	0.5000	96	4.5%	−0.01 [−0.30; 0.28]
Lexow et al. 2022	0.41	1.4400	96	1.26	1.2700	146	4.7%	−0.63 [−0.90; −0.37]
Rieckert et al. 2020	0.50	1.2600	1953	0.51	1.2400	1951	6.0%	−0.01 [−0.07; 0.05]
Tadrous et al. 2020	0.11	0.9000	249	0.10	0.9000	314	5.4%	0.01 [−0.16; 0.17]
van der Meer et al. 2018	0.31	0.2000	59	0.19	0.2000	77	4.0%	0.55 [0.20; 0.89]
Total (95% CI)			**3791**			**3988**	**49.1%**	**−0.02 [−0.21; 0.18]**
Heterogeneity: $Tau^2 = 0.0514$; $Chi^2 = 38.23$, df = 9 (P < 0.01); $I^2 = 76\%$								
subgroup = explicit								
Etherton−Beer et al. 2023	0.00	2.0000	102	0.00	2.0000	100	4.6%	0.00 [−0.28; 0.28]
Frankenthal et al. 2014	0.80	1.3000	160	1.30	2.4000	146	5.0%	−0.26 [−0.49; −0.04]
Holland et al. 2023	1.55	1.4400	230	1.26	1.2700	219	5.3%	0.21 [0.03; 0.40]
Jungo et al. 2023	0.20	0.5000	123	0.20	0.5000	125	4.8%	0.00 [−0.25; 0.25]
Juola et al. 2015	1.45	2.1000	118	2.38	2.1000	109	4.7%	−0.44 [−0.70; −0.18]
Kua et al. 2020	0.10	0.0800	221	0.08	0.0800	221	5.3%	0.28 [0.09; 0.47]
Mahlknecht et al. 2021	0.23	0.2000	281	0.30	0.2000	257	5.4%	−0.32 [−0.49; −0.15]
Patterson et al. 2010	2.15	2.0000	129	1.50	1.4000	124	4.8%	0.37 [0.12; 0.62]
Potter et al. 2016	4.91	4.0000	45	2.96	2.7000	48	3.5%	0.57 [0.16; 0.99]
Weber et al. 2008	0.05	0.0500	20	0.05	0.0500	20	2.3%	0.00 [−0.62; 0.62]
Wouters et al. 2017	0.26	0.2000	193	0.23	0.2000	193	5.2%	0.16 [−0.04; 0.35]
Total (95% CI)			**1622**			**1562**	**50.9%**	**0.04 [−0.16; 0.25]**
Heterogeneity: $Tau^2 = 0.0749$; $Chi^2 = 61.13$, df = 10 (P < 0.01); $I^2 = 84\%$								
**Total (95% CI)**			**5413**			**5550**	**100.0%**	**0.01 [−0.12; 0.14]**

Heterogeneity: $Tau^2 = 0.0606$; $Chi^2 = 99.94$, df = 20 (P < 0.01); $I^2 = 80\%$
Test for subgroup differences: $Chi^2 = 0.22$, df = 1 (P = 0.64)

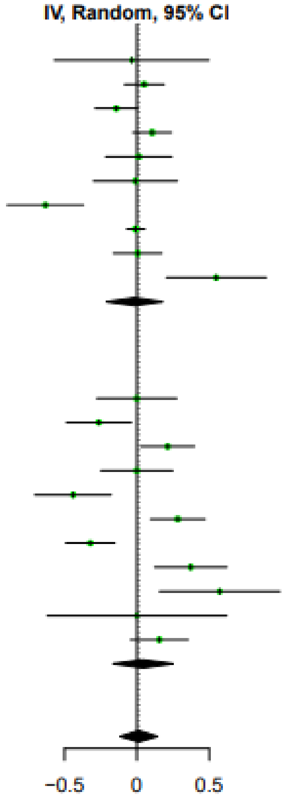

© 2025 Persaud N et al. *JAMA Network Open*.

eFigure 5. Quality-of-Life Effects of Potentially Inappropriate Prescribing Interventions

Study or Subgroup	Experimental Mean	Experimental SD	Experimental Total	Control Mean	Control SD	Control Total	Weight	Std. Mean Difference IV, Random, 95% CI
subgroup = implicit								
Auvinen et al. 2020	0.57	0.2900	258	0.56	0.2700	254	3.3%	0.04 [−0.14; 0.21]
Bosch−Lenders et al. 2021	6.70	6.9700	302	6.80	6.9700	302	3.3%	−0.01 [−0.17; 0.15]
Del Cura−González et al. 2022	0.76	0.2130	266	0.78	0.1820	263	3.3%	−0.09 [−0.26; 0.08]
Hanlon et al. 1996	37.40	1.6000	105	35.20	1.7000	103	2.9%	1.33 [1.03; 1.63]
Johansson et al. 2023	0.62	0.2700	24	0.47	0.3100	24	2.0%	0.51 [−0.07; 1.08]
Junius−Walker et al. 2021	0.54	0.3000	208	0.53	0.3000	199	3.2%	0.03 [−0.16; 0.23]
Köberlein−Neu et al. 2016	38.26	6.6000	105	38.47	6.6000	105	3.0%	−0.03 [−0.30; 0.24]
Kornholt et al. 2022	0.64	0.2920	196	0.64	0.2700	212	3.2%	−0.01 [−0.21; 0.18]
Lenaghan et al. 2007	0.57	0.2600	56	0.56	0.2600	49	2.6%	0.04 [−0.35; 0.42]
Lin et al. 2018	1.05	0.1600	45	0.83	0.1800	62	2.5%	1.27 [0.85; 1.69]
Liou et al. 2021	54.60	24.6000	42	62.10	24.4000	38	2.4%	−0.30 [−0.74; 0.14]
McCarthy et al. 2022	0.52	0.3820	177	0.46	0.3570	167	3.2%	0.16 [−0.05; 0.38]
Moga et al. 2017	65.60	13.2000	25	62.20	16.5000	24	2.0%	0.22 [−0.34; 0.79]
Muth et al. 2018	74.80	23.4000	202	72.80	25.1000	192	3.2%	0.08 [−0.12; 0.28]
Rieckert et al. 2018	36.73	9.4400	1775	36.32	9.1100	1774	3.5%	0.04 [−0.02; 0.11]
Romskaug et al. 2020	0.68	0.1860	81	0.62	0.2160	81	2.9%	0.27 [−0.04; 0.58]
Rougehead et al. 2022	0.20	0.3390	107	0.16	0.3290	112	3.0%	0.12 [−0.15; 0.38]
Schäfer et al. 2018	0.68	0.3200	132	0.70	0.2800	135	3.1%	−0.07 [−0.31; 0.17]
Verdoorn et al. 2019	0.73	0.2000	266	0.74	0.1800	261	3.3%	−0.05 [−0.22; 0.12]
Zechmann et al. 2020	1.46	0.3500	97	1.43	0.3300	157	3.0%	0.09 [−0.16; 0.34]
Total (95% CI)			**4469**			**4514**	**58.8%**	**0.16 [−0.02; 0.35]**
Heterogeneity: $Tau^2 = 0.1283$; $Chi^2 = 115.92$, df = 19 ($P < 0.01$); $I^2 = 84\%$								
subgroup = explicit								
Beer et al. 2011	75.00	25.0000	15	66.00	26.0000	20	1.7%	0.34 [−0.33; 1.02]
Bogaerts et al. 2024	−1.20	15.6000	52	−6.40	13.3000	66	2.7%	0.36 [−0.01; 0.73]
Callegari et al. 2022	23.11	8.7200	63	22.74	7.6400	63	2.7%	0.04 [−0.30; 0.39]
Clyne et al. 2015	23.60	3.3400	70	24.00	3.3400	69	2.8%	−0.12 [−0.45; 0.21]
Etherton−Beer et al. 2023	0.51	0.3200	102	0.58	0.2900	100	3.0%	−0.23 [−0.50; 0.05]
Frankenthal et al. 2014	33.10	8.1000	160	33.00	8.3000	146	3.1%	0.01 [−0.21; 0.24]
Holland et al. 2023	0.26	0.3500	230	0.21	0.3300	219	3.2%	0.15 [−0.04; 0.33]
Jungo et al. 2023	0.10	0.2290	123	0.10	0.2290	125	3.1%	0.00 [−0.25; 0.25]
Olsson et al. 2012	54.92	15.3700	72	56.00	17.0000	34	2.5%	−0.07 [−0.48; 0.34]
Pitkälä et al. 2014	−0.04	0.0730	78	−0.07	0.0790	72	2.8%	0.45 [0.12; 0.77]
Potter et al. 2016	−11.00	17.0000	47	7.00	15.0000	48	2.4%	−1.11 [−1.55; −0.68]
Rankin et al. 2022	0.72	0.1920	21	0.77	0.1570	17	1.8%	−0.28 [−0.92; 0.37]
Richter et al. 2019	33.10	8.4000	179	34.00	8.4000	246	3.2%	−0.11 [−0.30; 0.09]
Rudolf et al. 2021	67.60	6.2600	319	67.00	8.5500	305	3.3%	0.08 [−0.08; 0.24]
Wouters et al. 2017	0.41	0.2700	97	0.33	0.2600	80	2.9%	0.30 [0.00; 0.60]
Total (95% CI)			**1628**			**1610**	**41.2%**	**−0.00 [−0.19; 0.19]**
Heterogeneity: $Tau^2 = 0.0796$; $Chi^2 = 49.32$, df = 14 ($P < 0.01$); $I^2 = 72\%$								
**Total (95% CI)**			**6097**			**6124**	**100.0%**	**0.09 [−0.04; 0.23]**
Heterogeneity: $Tau^2 = 0.1105$; $Chi^2 = 166.31$, df = 34 ($P < 0.01$); $I^2 = 80\%$								
Test for subgroup differences: $Chi^2 = 1.70$, df = 1 ($P = 0.19$)								

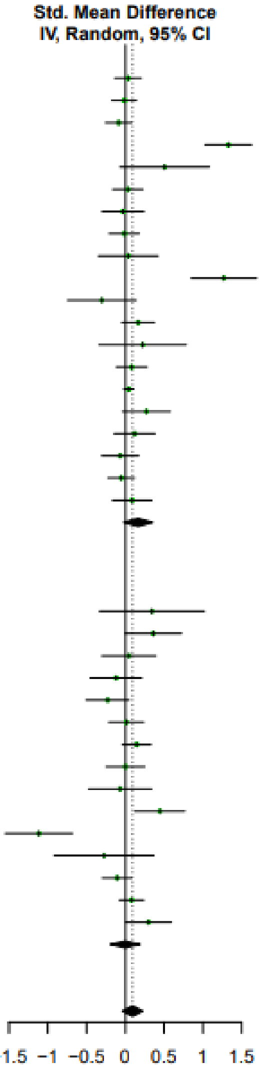

© 2025 Persaud N et al. *JAMA Network Open*.

eFigure 6. Medical Visits Effects of Potentially Inappropriate Prescribing interventions

Study or Subgroup	Experimental Mean	Experimental SD	Experimental Total	Control Mean	Control SD	Control Total	Weight	Std. Mean Difference IV, Random, 95% CI
subgroup = implicit								
Del Cura−González et al. 2022	7.00	5.1900	243	7.00	4.4400	250	9.2%	0.00 [−0.18; 0.18]
García−Gollarte et al. 2014	2.76	2.2800	287	2.82	2.6600	279	10.6%	−0.02 [−0.19; 0.14]
Herrinton et al. 2023	11.40	9.8500	1237	11.30	9.8300	1233	46.3%	0.01 [−0.07; 0.09]
Liou et al. 2021	4.30	3.1000	42	4.20	4.0000	38	1.5%	0.03 [−0.41; 0.47]
McCarthy et al. 2022	7.00	6.6000	177	6.20	3.9000	167	6.4%	0.15 [−0.07; 0.36]
Schäfer et al. 2017	4.60	3.8000	132	4.10	3.5000	135	5.0%	0.14 [−0.10; 0.38]
Syafhan et al. 2021	2.50	3.1000	169	2.80	2.9000	169	6.3%	−0.10 [−0.31; 0.11]
Total (95% CI)			**2287**			**2271**	**85.4%**	**0.01 [−0.04; 0.07]**
Heterogeneity: $Tau^2 = 0$; $Chi^2 = 3.83$, df = 6 (P = 0.70); $I^2 = 0\%$								
subgroup = explicit								
Campins et al. 2017	30.90	18.3000	252	29.80	16.0000	251	9.4%	0.06 [−0.11; 0.24]
Clyne et al. 2016	12.20	10.0000	70	11.70	10.0000	69	2.6%	0.05 [−0.28; 0.38]
Lenander et al. 2014	1.60	1.5200	75	1.40	1.0200	66	2.6%	0.15 [−0.18; 0.48]
Total (95% CI)			**397**			**386**	**14.6%**	**0.08 [−0.03; 0.18]**
Heterogeneity: $Tau^2 = 0$; $Chi^2 = 0.24$, df = 2 (P = 0.89); $I^2 = 0\%$								
**Total (95% CI)**			**2684**			**2657**	**100.0%**	**0.02 [−0.02; 0.07]**
Heterogeneity: $Tau^2 = 0$; $Chi^2 = 4.73$, df = 9 (P = 0.86); $I^2 = 0\%$								
Test for subgroup differences: $Chi^2 = 3.30$, df = 1 (P = 0.07)								

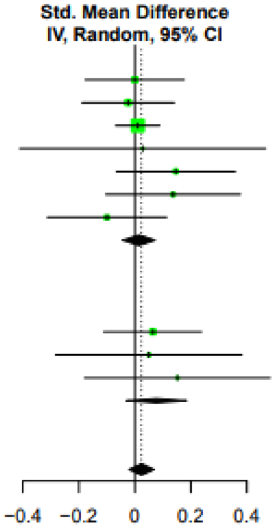

© 2025 Persaud N et al. *JAMA Network Open*.

eFigure 7. Emergency Department Visit Effects of Potentially Inappropriate Prescribing

Study or Subgroup	Experimental Events	Experimental Total	Control Events	Control Total	Weight	Risk Ratio IV, Random, 95% CI
subgroup = explicit						
Almutairi et al. 2023	5	66	4	91	0.4%	1.72 [0.48; 6.17]
subgroup = implicit						
Boyd et al. 2024	25	179	33	203	3.0%	0.86 [0.53; 1.39]
Del Cura−González et al. 2022	1	267	1	264	0.1%	0.99 [0.06; 15.73]
Elliot et al. 2017	13	57	17	53	1.8%	0.71 [0.38; 1.32]
Haag et al. 2015	1	11	1	11	0.1%	1.00 [0.07; 14.05]
Herrinton et al. 2023	502	1237	493	1233	73.6%	1.01 [0.92; 1.12]
Junius et al. 2021	41	167	33	172	4.1%	1.28 [0.85; 1.92]
Krska et al. 2001	6	168	8	164	0.6%	0.73 [0.26; 2.06]
Lenaghan et al. 2007	20	68	21	66	2.6%	0.92 [0.55; 1.54]
Schmidt et al. 2016	78	353	83	394	9.1%	1.05 [0.80; 1.38]
Verdoon et al. 2019	46	315	44	314	4.6%	1.04 [0.71; 1.53]
**Total (95% CI)**		**2822**		**2874**	**99.6%**	**1.01 [0.95; 1.08]**
Heterogeneity: $Tau^2 = 0$; $Chi^2 = 3.57$, df = 9 (P = 0.94); $I^2 = 0\%$						
**Total (95% CI)**		**2888**		**2965**	**100.0%**	**1.02 [0.96; 1.08]**

Heterogeneity: $Tau^2 = 0$; $Chi^2 = 4.24$, df = 10 (P = 0.94); $I^2 = 0\%$

Test for subgroup differences: $Chi^2 = 0.67$, df = 1 (P = 0.41)

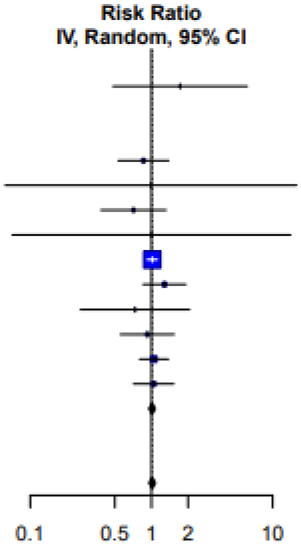

© 2025 Persaud N et al. *JAMA Network Open*.
