# Supplementary material for: Interventions to Address Potentially Inappropriate Prescribing for Older Primary Care Patients: A Systematic Review and Meta-Analysis
Source: JAMA Netw Open. 2025 Jun 27;8(6):e2517965. doi: 10.1001/jamanetworkopen.2025.17965 (PMC12205406; doi:10.1001/jamanetworkopen.2025.17965)
Supplement: Supplement 2. — Data Sharing Statement [file jamanetwopen-e2517965-s002.pdf]

Data Sharing Statement

Persaud. Interventions to Address Potentially Inappropriate Prescribing for Older Primary Care Patients. *JAMA Netw Open*. Published June 27, 2025.
doi:10.1001/jamanetworkopen.2025.17965

Data
Data available: No
